# Determining Electric Fields in Thunderclouds With the Radiotelescope LOFAR

**DOI:** 10.1029/2019JD031433

**Published:** 2020-04-22

**Authors:** T. N. G. Trinh, O. Scholten, S. Buitink, U. Ebert, B. M. Hare, P. R. Krehbiel, H. Leijnse, A. Bonardi, A. Corstanje, H. Falcke, T. Huege, J. R. Hörandel, G. K. Krampah, P. Mitra, K. Mulrey, A. Nelles, H. Pandya, J. P. Rachen, L. Rossetto, C. Rutjes, S. ter Veen, T. Winchen

**Affiliations:** ^1^ Department of Physics, School of Education Can Tho University Campus II Can Tho Vietnam; ^2^ KVI‐Center for Advanced Radiation Technology University of Groningen Groningen The Netherlands; ^3^ Inter University Institute for High Energies Vrije Universiteit Brussel Brussels Belgium; ^4^ Astrophysical Institute Vrije Universiteit Brussel Brussels Belgium; ^5^ Department of Astrophysics/IMAPP Radboud University Nijmegen Nijmegen The Netherlands; ^6^ Center for Mathematics and Computer Science (CWI) Amsterdam The Netherlands; ^7^ Department of Applied Physics Eindhoven University of Technology (TU/e) Eindhoven The Netherlands; ^8^ Langmuir Laboratory for Atmospheric ResearchGeophysical Research Center New Mexico Institute of Mining and Technology Socorro NM USA; ^9^ Royal Netherlands Meteorological Institute (KNMI) De Bilt The Netherlands; ^10^ Nikhef Amsterdam The Netherlands; ^11^ Netherlands Institute of Radio Astronomy (ASTRON) Dwingeloo The Netherlands; ^12^ Max‐Planck‐Institut für Radioastronomie Bonn Germany; ^13^ Institut für Kernphysik Karlsruhe Institute of Technology (KIT) Karlsruhe Germany; ^14^ Erlangen Centre for Astroparticle Physics Friedrich‐Alexander‐Univeristät Erlangen‐Nürnberg Erlangen Germany; ^15^ DESY Zeuthen Germany

## Abstract

An analysis is presented of electric fields in thunderclouds using a recently proposed method based on measuring radio emission from extensive air shower events during thunderstorm conditions. This method can be regarded as a tomography of thunderclouds using cosmic rays as probes. The data cover the period from December 2011 till August 2014. We have developed an improved fitting procedure to be able to analyze the data. Our measurements show evidence for the main negative‐charge layer near the −10° isotherm. This we have seen for a winter as well as for a summer cloud where multiple events pass through the same cloud and also the vertical component of the electric field could be reconstructed. On the day of measurement of some cosmic‐ray events showing evidence for strong fields, no lightning activity was detected within 100 km distance. For the winter events, the top heights were between 5 and 6 km, while in the summer, typical top heights of 9 km were seen. Large horizontal components in excess of 70 kV/m of the electric fields are observed in the middle and top layers.

## Key Points

- Routine nonintrusive determination of atmospheric electric fields can be done from the radio footprint of cosmic‐ray air showers at LOFAR
- Large components of the electric fields are measured in clouds with appreciable radar reflectivity as well as without lightning activity
- Finding the main negative‐charge layer near the −10 isotherm for the cases where the 3‐D field was reconstructed agrees with literature

## 1 Introduction

Lightning is a very interesting phenomenon, but a detailed understanding of the process is still missing (Dwyer & Uman, 2014). Even the most basic of lightning processes, such as initiation (Marshall et al., 1995) and propagation (Hill et al., 2012), are difficult to understand due to the lack of knowledge of electric fields inside thunderstorms. It is argued that understanding thunderstorm electric fields is also critical for understanding high‐energy lightning phenomena, such as gamma‐ray glows (Kochkin et al., 2017), terrestrial gamma‐ray flashes (TGFs) (e.g.,  Carlson et al., 2010; Dwyer, 2012; Hare et al., 2016), neutron emission (Shah et al., 1985), and the recently discovered gamma‐ray afterglow (Enoto et al., 2017; Rutjes et al., 2017). In addition, clouds that do not produce lightning can still have strong electric fields, which can be dangerous due to the potential for airplanes to trigger lightning (Merceret et al., 2008).

Measuring electric fields is thus an important task. Much expertise has been built with measuring these fields using aircrafts (Jones et al., 1993) and balloons (Marshall et al., 1995). These methods have given important contributions because they measure the local fields rather accurately. The measurements are, however, not easy to perform as balloons have to be launched in adverse weather conditions. In addition, when they enter cloud regions with strong fields, the balloon with the attached equipment may trigger a lightning discharge that ends the mission. It is thus interesting to have nonintrusive methods for measuring the fields. One such method was proposed in Carlson and Inan (2008) using the electrically induced birefringence (Kerr) effects on natural sky polarization. In this work, we elaborate another nonintrusive method, recently introduced in Schellart et al. (2015), that uses the infrastructure offered by Low‐Frequency Array (LOFAR) (van Haarlem et al., 2013), a modern software telescope consisting of thousands of antennas distributed over a large area, primarily constructed for astronomical observations (see section 2.1). The method is based on analyzing the radio footprint of cosmic ray‐induced air showers. We call this cosmic‐ray cloud tomography (CRCT). To be able to perform CRCT, one needs the dense distribution of antennas as can only be found at the LOFAR core. As such, this offers a unique opportunity to perform these measurements, which are made automatically (unavoidably) whenever LOFAR measures cosmic rays. This opens the possibility for random testing of cloud electric fields in thunderclouds of the temperate zone. These have not been studied much as they are small, in stark contrast to the of impressive supercell thunderclouds as occur in tropical areas. As these storms occur in densely populated areas, it is certainly of interest to study them. We report here on the initial period of the cosmic‐ray program (till August 2014), which is why the statistics is still small.

It should be noted that cosmic rays have also been used at other facilities to learn about very‐strong atmospheric electric fields. At the GRAPES‐3 muon telescope located in Ooty, India, strong variations in muons have been measured that have been used to determine potential differences in thunderclouds of 1.3 GV (Hariharan et al., 2019). At the Pierre Auger Observatory, ring‐like structures in the counting rate of the surface particle detectors have been observed. These rings have diameters of the order of a few kilometers that can be explained as due to strong atmospheric electric fields of the order of 500 kV/m extending over a distance of a kilometer (Colalillo, 2019). In our observations with the LOFAR radio telescope, we measured fields of the order of 100 kV/m, more in line with expectations for smaller thunderclouds as are typical for the temperate climate zone of the Netherlands.

In the LOFAR measurements, we are primarily sensitive to the component of the atmospheric electric field that is perpendicular to the shower axis due to the dynamics in a cosmic ray‐induced air shower (Trinh et al., 2016). The component parallel to the axis cannot be determined, but the height dependence of the perpendicular component, the magnitude and direction, can be obtained. Since the cosmic ray makes on average an angle of 30° with the vertical, there is sensitivity to the vertical component. As discussed in section 3.4, the full 3‐D electric field vector can be reconstructed when at least two cosmic rays pass through the same cloud at different angles.

We find strong horizontal components of the field that are consistent with the rocket observations presented in Winn et al. (1974) and which are much larger than what is reported from airplane observations (Mo et al., 2002). These airplane observations have taught that the fields may have any orientation where the vertical component is of similar, be it somewhat larger, magnitude as the horizontal. The fact that horizontal and vertical components may have a similar value is in agreement with what we find in the present work and which may be typical for the smaller‐size thunderclouds seen in the temperate zone.

In section 2, we describe CRCT, the method of using cosmic ray‐induced air showers in tomographic imaging of the electric fields in clouds. Although the procedure follows our earlier work (Trinh et al., 2017), we have made considerable improvements in the fitting procedure to extract the parameters of the atmospheric electric fields as discussed in section 2.3. This makes it practical to use this method in a more routinely manner. The analysis of 11 so‐called thunderstorm cosmic‐ray events measured during a two‐and‐a‐half‐year period is presented in section 2.4. Some of the events occur within 15 min from each other, giving the possibility to have multiple measurements of the same cloud. This, in principle, allows for a full reconstruction of the three components of the fields inside a single cloud. In practice, due to the very dynamic environment in the cloud and its small extent, the charge structure may change over this short time span. It is tempting to interpret our measurements of the electric field in terms of horizontal charge layers at certain heights. This is not an easy endeavor since we have only partial sensitivity to the vertical components, as discussed in section 3.1. We correlate our observations with weather radar reflectivity images (ADAGUC, 2018) and lightning observations (KNMI, 2018). We confirm that also for smaller thunderclouds, as are common in the temperate zone, the height of the main negative‐charge layer lies close to the −10° isotherm. We also find that clouds that do not show any lightning activity but show moderately strong radar reflectivity may carry strong electric fields, which is consistent with the findings in Dye et al. (1989). These clouds will contribute to the Global Electric Circuit (Bering‐III et al., 1998; Williams & Heckman, 1993) since the lower charge layers will discharge through rain to the surface of Earth.

## 2 Cosmic‐Ray Tomography of Cloud Electric Fields

When cosmic rays of very high energy (∼ 10^17^ eV) penetrate Earth's atmosphere, they create a shower of secondary particles moving with ultrarelativistic velocities toward Earth's surface. Most of the particles in the shower are photons, electrons, and positrons. Since all particles have almost the same velocity, they move as a pancake. As explained in section 2.2, the charged particles are deflected sideways from the shower direction due to the Earth magnetic field as well as due to atmospheric electric fields. This induces a net current that, due to the relativistic velocities, emits a very short, of the order of nanoseconds, radio pulse. Again, due to the relativistic velocities, this pulse is beamed in the forward direction and thus only measurable in a limited area around the point of impact of the cosmic ray on the ground. Important for this work is that the distribution over the ground plane of the intensity and polarization (the radio footprint) of this short pulse acts as a finger print for the height dependence of the induced current in the shower front. Thus, the cosmic rays act as a tomographic probe for the atmospheric electric fields.

**Figure 1:**
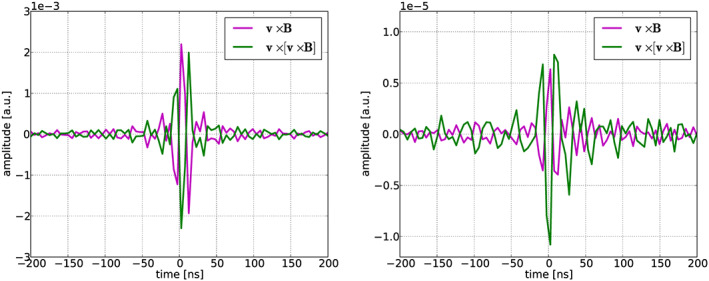
The simulated radio pulse, including the effects of the band‐pass filter, (left) is compared to measured pulse (right) for the two polarization directions of an antenna from Event #8. The vertical scale of the model calculation is in arbitrary units (left) and the digitizer units (right) for the measurement.

**Figure 2:**
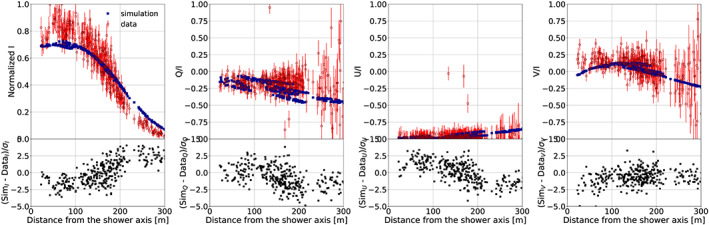
The results for normalized Stokes parameters (filled blue dots) calculated with CoREAS for Event #6, using the field configuration given in Table 1, are compared to LOFAR data (open red circles). Bottom panel shows the difference between calculation and data normalized by *σ*, the one standard deviation error.

**Figure 3:**
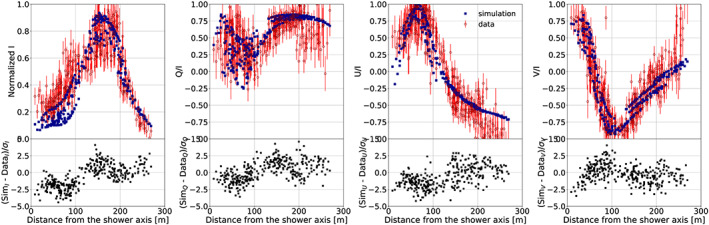
Same as Figure 2 for Event #7.

Since the radio footprint, due to the effects of atmospheric electric fields, can be rather intricate, it is necessary to have a high density of antennas to measure the intensity of the radio signal as function of distance to the core of the shower (preferably antennas at about 10 m separation and up to 300 m from the shower core) in addition to the complete polarization profile of the signal that includes circular polarization. Thus, for such measurements, LOFAR (van Haarlem et al., 2013), explained further in section 2.1, is ideal. Since the pulses are very short, the measured signals are bandwidth limited for which reason it is generally sufficient to determine the pulse strength and the polarization in terms of Stokes parameters. An example of a measured pulse is shown in Figure 1 where the oscillations are due to the band‐pass filter (30–80 MHz) and not due to the structure in the emitting current. The radio footprint can be plotted as the values of the different Stokes parameters versus distance to the shower axis, as in Figures 2 and 3, which is the basic information we use in the subsequent analysis.

It is still a major challenge to reconstruct the structure of the atmospheric electric fields from the measured radio footprint. Given a configuration of atmospheric electric fields, it is reasonably straightforward to calculate the radio footprint and to solve the inverse problem we have developed the approach discussed in section 2.3. This is based on chi‐square fitting of the parameters of a simplified atmospheric field configuration to reproduce the measured radio footprint. To present some examples, selected cases are discussed in some more detail in section 2.4. The final results for all cosmic‐ray events that could be analyzed are given in section 3 together with an interpretation in terms of charge layers.

### 2.1 LOFAR and Data Analysis

Data were recorded with LOFAR, a radio telescope with its core in the northern part of the Netherlands and with many remote stations across Europe. The antennas of LOFAR are grouped into stations. Each station contains 96 low‐band antennas (LBAs; 10–90 MHz) and 48 high‐band antennas (HBAs; 110–240 MHz). The densest concentration of antennas, called the “Superterp,” is located near Exloo, in Drenthe in the Netherlands. The Superterp has a diameter of ∼320 m and contains six stations. For our observations, the received signal, a short pulse, is sampled every 5 ns and stored for 5 s on ring buffers for each active antenna. These buffers are read out once a trigger signal is obtained from a particle detector array, the LOFAR Radboud air shower Array (LORA) consisting out of 20 scintillator counters with an area of 1 m^2^ each, suitable for detecting air showers with a primary energy in excess of 2×10^16^ eV (Thoudam et al., 2014). Data for the present analysis are taken from LBAs mainly at the Superterp.

We use the following criterion to select good “thunderstorm” cosmic‐ray events. First, as shown in Schellart et al. (2015), the linear polarization in thunderstorm events is very different from that in fair‐weather events. Second, the events should have radio signals recorded in at least four LBA stations, and there should be at least one station within 100 m from the shower axis receiving signals. Third, the mean relative uncertainty of the intensity should be less than 30*%*; otherwise, the uncertainties of the data are too large to draw any conclusions. Fourth, since the position where the core of the air shower hits the ground (core position) is very essential in fitting, the event should have an energy deposit larger than 100 MeV in at least one LORA scintillator counter.

For this work, we have analyzed data from December 2011 to August 2014. During this period, there were 31 measured thunderstorms events from which 11 events obey all aforementioned quality conditions. The data were processed in an offline analysis (Schellart et al., 2013) where the arrival direction and the energy of air showers were estimated. The data are Fourier transformed and filtered to the interval from 30 to 80 MHz since below 30 MHz and above 80 MHz, the spectrum is dominated by anthropogenic sources (such as radio and TV stations). In addition, for each antenna, the real‐valued Stokes parameters, expressed as

(1) $$I=\frac{1}{n}\overset{n-1}{\underset{i=0}{\sum }}\left({\left|\varepsilon_{i,v\times B}\right|}^{2}+{\left|\varepsilon_{i,v\times \left(v\times B\right)}\right|}^{2}\right),$$

(2) $$Q=\frac{1}{n}\overset{n-1}{\underset{i=0}{\sum }}\left({\left|\varepsilon_{i,v\times B}\right|}^{2}-{\left|\varepsilon_{i,v\times \left(v\times B\right)}\right|}^{2}\right),$$

(3) $$U+iV=\frac{2}{n}\overset{n-1}{\underset{i=0}{\sum }}\left(\varepsilon_{i,v\times B}\varepsilon_{i,v\times \left(v\times B\right)}^{*}\right),$$

are calculated (Schellart et al., 2014) where **v** denotes the direction of the cosmic ray and **B** that of the Earth magnetic field. Since the emission is strongly beamed in the direction **v** along the shower, **v**×**B** and $v\;\times \;\left(v\times B\right)$ are thus two polarization directions orthogonal to the direction of the radio wave and were chosen because of the physics under fair‐weather circumstances as outlined in the following section. *ε*_*i*_ is the complex‐valued signal radiation field where *i* denotes the sample number (at 2×10^8^ samples per second). The radiation fields are recovered from the measured voltages by inverting the antenna calibration. The summation is performed over *n*=11 samples, centered around the peak of the pulse. Stokes *I* is the intensity of the radio emission. Stokes *Q* and *U* are used to derive the linear polarization angle

(4) $$\psi =\frac{1}{2}\tan^{-1}\left(\frac{U}{Q}\right)\;,$$

and Stokes *V* represents the circular polarization.

### 2.2 Basic Principle of Radio Emission

When a high‐energy cosmic ray enters the Earth's atmosphere, a collision with an air molecule will generate many secondary particles. At sufficiently high energy, these secondary particles will subsequently collide with air molecules and create an avalanche of particles in the atmosphere called an extensive air shower (EAS). In the plasma at the shower front, there are many (order of 10^6^, depending on the energy of the cosmic ray) electrons and positrons. Under fair‐weather conditions, these electrons and positrons are deflected in opposite directions by the Lorentz force induced by the geomagnetic field. This generates a transverse current pointing in the direction of the Lorentz force, and the changing current (changing with height in the atmosphere) emits radio‐frequency radiation (Scholten et al., 2008). This signal is linearly polarized along the direction of the Lorentz force, the **v**×**B** direction, where **v** is the direction of the shower and **B** is the Earth's magnetic field. At LOFAR, we have *B*=49.5μT oriented at a zenith angle of 22.19° southward. An excess of negative charge is built up in the shower front from the electrons that are knocked out of atmospheric molecules by interactions with shower particles. Due to this charge excess, also, a radio pulse is emitted (Askaryan, 1962; de Vries et al., 2010), which is linearly polarized but oriented radially to the shower axis.

Atmospheric electric fields affect the induced electric currents in air showers and thus the radiation from them (Trinh et al., 2016). The electric field component parallel to the shower axis, **E**_‖_, increases the number of electrons or positrons, depending on its sign. However, the particles generated by **E**_‖_ have low energies, and thus, they trail far behind the shower front. For this reason, the radio emission of the additional charged particles contributes coherently in the low‐frequency regime of less than 10 MHz, well below the frequency range of the LOFAR LBAs, which ranges from 30 to 80 MHz. As a result, LOFAR is not sensitive to **E**_‖_. The technique could be made more sensitive to **E**_‖_ by adding antennas that are sensitive to the 1 to 10 MHz band (see Trinh et al., 2016). For moderate‐to‐high **E**_‖_ fields at low altitude above the ground, one would expect a clear increase in the count rate of particle detectors on the ground (see, e.g., Chilingarian et al., 2014; Cramer et al., 2017). We have searched for enhanced count rates in the LORA detectors (Wempe, 2019) but not found any, which is supporting the fact that the fields at low altitude are relatively weak. Only when **E**_‖_ is strong enough to generate avalanches, as is presumably the case in Colalillo (2019), a clear signal would be seen in LORA and LOFAR. Its absence implies that the fields stay below this limit of about 400 kV/m.

The electric field perpendicular to the shower axis, **E**_⊥_, does not change the number of particles but changes the direction and the strength of the force acting on the particles. As a result, the direction and the magnitude of the current in the shower front changes, and thus, the emitted radio signals do change as well. A strong circular polarization in the emitted radio signal is induced when the direction of the electric field varies with height (Trinh et al., 2017), which results in a completely different emission pattern as for fair‐weather conditions (Scholten et al., 2016).

As a result, very different intensity and polarization footprints are to be expected for the radio emission of an air shower event measured while there is a cloud overhead with a considerable amount of electrification (which occurs not only during a thunderstorm but nevertheless is called “thunderstorm” event), as compared to an air shower recorded during fair weather (a so‐called fair‐weather event) (Schellart et al., 2015). Because of its large effect on the radio footprint, we can determine **E**_⊥_ in clouds. Our sensitivity drops considerably for heights above about 8 km and lower than about 1 km since at these high and low altitudes, the number of shower particles is small, and thus, the radio signals emitted from them are negligible. For this reason, we have taken the approach to search for solutions where the upper height for the field configuration is taken close to the measured echo top heights (i.e., the maximum altitude where the radar reflectivity factor exceeds 7 dBZ), which is a very good indication of the cloud top height. When a considerable better fit could be obtained by assuming an electric field above the clouds, we deviated from this rule. This situation occurred for Events #1, #4, and, to a certain extent, #3, while for the others, we could achieve satisfactory results by assuming a vanishing field above the clouds (see Table 1).

**Table 1:** The Left Part of the Table Shows the Event ID, the UTC Time of Measurement, and the Direction of the 10 Showers That Are Analyzed

	UTC date	*θ*	*ϕ*	*h*_0_	*h*_−10_	*h*_top_		*h*_*i*_	*E*_*i*_	*α*_*i*_	*E*_**v**×**z**_	$E_{v\times \left(v\times z\right)}$
ID	Time	(°)	(°)	(km)	(km)	(km)	*i*	(km)	(kV/m)	(°)	(kV/m)	(kV/m)
1	14/12/2011	39.4	144.8	0.7	2.3	5.9	No stable fit					
	21:02:27											
2	14/12/2011	14.1	134.0	0.7	2.3	4.8	1	7.5	53	−171	42.4	32.2
	21:10:01						2	2.0	82	13	−62.0	−53.4
3	14/12/2011	24.4	333.0	0.7	2.3	5.2	1	6.1	52	−170	35.9	−37.7
	21:14:34						2	5.3	63	−62	29.0	55.4
							3	2.5	0	7	−0.1	0.1
4	26/04/2012	22.2	129.0	1.5	3.4	6.1	0	15.0	30	158	23.0	−0.4
	15:22:33						1	7.7	43	56	−9.7	−42.0
							2	3.7	−31	−5	30.1	8.8
							3	2.2	22	−95	−5.9	20.9
5	28/07/2012	22.3	2.2	3.6	5.6	7.2	1	7.0	72	−53	10.9	71.7
	02:20:21						2	5.5	104	−106	92.3	48.9
							3	3.2	16	−168	13.1	−8.9
6	26/08/2012	22.8	143.8	2.5	4.2	8.7	1	9.1	57	−63	−47.3	32.3
	13:52:23						2	4.0	3	−154	1.9	2.7
							3	1.2	4	−20	−4.3	−0.6
7	26/08/2012	17.6	309.5	2.5	4.2	8.9	1	5.8	30	−29	14.1	26.2
	14:02:56						2	3.4	83	180	1.0	−82.5
							3	1.7	13	30	−6.9	11.6
8	26/08/2012	24.8	308.7	2.5	4.2	9.1	1	7.2	42	−97	39.5	14.7
	14:28:19						2	3.7	73	−137	68.9	−24.9
							3	3.0	24	69	−23.7	−2.5
9	30/12/2012	15.6	304.0	0.8	2.2	5.3	1	4.6	34	116	−33.3	−7.1
	12:38:37						2	1.5	33	21	−4.0	32.5
10	26/07/2013	15.5	40.2	3.8	5.7	11.0	1	7.4	88	80	−57.9	−66.8
	12:17:26						2	5.0	92	−102	62.3	67.3
							3	3.5	68	−136	65.7	15.6
11	27/06/2014	14.6	238.6	2.5	4.2	6.4	1	6.4	104	41	92.3	−47.6
	14:44:03						2	4.5	60	−28	−6.1	−59.9
							3	3.0	4	−115	−3.6	0.2

*Note*. The columns labeled *h*_0_ and *h*_−10_ show the altitude of the 0° and −10° isotherms obtained from GDAS (GDAS, 2018) data. The echo top heights are given in column *h*_top_. The following columns show the height of the top height of the layer and the magnitude and angle *α* (w.r.t.  *e*_**v**×**B**_) of **E**_⊥_ as determined from the best fit (see the supporting information). Note that Event #2 has only two layers and Event #4 four layers. The last two columns are discussed extensively in section 3.3.

Unlike other electric field measurements, this technique is nonintrusive; that is, it does not disturb the electric fields in the cloud during the measurement and it can be regarded as a tomographic method. The cosmic ray‐induced air showers are used to probe the perpendicular electric field structure inside the clouds (Schellart et al., 2015) along its track. By combining the information from a few such observations, the full field can be reconstructed (see section 3.4).

Given an electric field configuration, the radio profile can readily be calculated. In contrast, the inverse problem is hard to solve. Thus, to extract the atmospheric electric field profile from the measured data, one needs to solve a complicated inverse problem. This is discussed in section 2.3, where we calculate the expected radio profile for a given electric field configuration and minimize the chi‐square deviation with the measured radio profile to obtain the best fitting electric field configuration.

### 2.3 Electric Field Reconstruction Technique

There are two microscopic models that accurately simulate the complete radiation field emitted from an EAS, ZHAires (Alvarez‐Muiz et al., 2012), and CoREAS (Huege et al., 2012), where CoREAS is a plug‐in for the shower simulation code CORSIKA (Heck et al., 1998). In the latter code, atmospheric electric fields are implemented by turning on the EFIELD option (Buitink et al., 2010). In these microscopic codes, a full air shower simulation is performed where for each particle in the shower, the radiation field is calculated, and these fields are summed at the position of each antenna.

For fair‐weather measurements of air showers, these codes are used to extract from the radio‐emission intensity footprint a single parameter, *X*_max_, the atmospheric depth where the number of secondary particles reaches a maximum in the EAS, as this pertains to the physics one needs to address in cosmic‐ray physics. To obtain the value of *X*_max_, the measured intensity footprint is fitted through a simple one‐dimensional grid search as presented in Buitink et al. (2014). For thunderstorm cosmic‐ray events, we will follow the same basic principle. However, in clouds, the electric field will depend on height. Thus, the transverse current in an air shower varies strongly with height because of changes in the strength and orientation of the atmospheric electric fields. This makes fitting considerably more difficult than for the fair‐weather case for two reasons. (1) To account for the orientations of the fields, it is necessary to consider besides intensity also polarization data or, equivalently, the full set of Stokes parameters. (2) The profile of the electric field needs to be parameterized, and a realistic parameterization easily requires more than eight parameters.

One thus needs an automatized chi‐square fitting procedure in a multidimensional parameter space (in practice, there are about 11 parameters that are searched). This precludes the use of microscopic calculations for two reasons. First, they are based on a Monte Carlo simulation where changing a single shower parameter will affect the development of the shower as a whole. A small change in input parameters thus does not correspond to a small change in the simulated radio profile. Second, these calculations use considerable computer resources because individual charged particles are traced. For these reasons, an analytic code called MGMR3D has been developed (Scholten et al., 2018) to amend this issue. MGMR3D calculates the radio footprint from Maxwell equations for a parameterized charge/current density for an EAS. The parametrization is based on the results of microscopic shower simulations where special attention is paid to the effects from atmospheric electric field on showers as have been studied extensively in Trinh et al. (2016). This model is not Monte Carlo based and requires little computing time. It has been shown that MGMR3D gives a good agreement with CoREAS for fair‐weather showers as well as for the cases where there are strong atmospheric electric fields (Scholten et al., 2018, 2019) to account for.

To make the problem tractable, we will use as the basis in this work a triple‐layered structure for the atmospheric electric field. This is inspired by the idealized structure of charge layers in (thunder) clouds where around the freezing level, one (usually) finds a positively charged layer, at −10 to −20 °C (Krehbiel, 1986) the main negative layer and near the top of the cloud another positive layer. In actual balloon measurements in well‐developed cumulonimbus clouds, one often observes much more intricate structures for the electric fields (Marshall et al., 1995; Stolzenburg et al., 2010) most probably due to the strongly turbulent cloud dynamics. A recent summer lightning flash mapped by LOFAR (Hare et al., 2019) shows evidence of a simple charge structure, with positive charge around 2‐ to 3‐km altitude and negative charge around 5‐km altitude.

In our parametrization of the electric fields, we will also use a layered structure. Each layer *i* is defined by *h*_*i*_, the altitude of the top of the layer, and the strength and direction of the component of the field in this layer that is perpendicular to the shower axis, **E**_⊥*i*_. The layers will be identified by indices 1, 2, and 3. The top layer, 1, thus has the field **E**_⊥1_ stretching between the heights *h*_1_ and *h*_2_. The bottom layer, often layer 3, with the field **E**_⊥3_ is between *h*_3_ and the ground. For simplicity, the electric field **E**_⊥_ is taken constant in each layer, and thus, our results should be interpreted as an average of the electric field in a particular layer. We used this simple‐layered parameterization in order to keep from having to fit too many free parameters. Any change in the field over distances smaller than about 500 m has only very minor effects (Trinh et al., 2016) on the emitted radio signal and thus cannot be resolved. In the actual calculations, the top height, *h*_1_, is taken close to the echo top heights that have been determined from radar reflection data. This is done because our method has limited sensitivity to the fields at large heights. In searching for the electric field conditions, we have initially fixed the height of the top most layer to that of the echo top height (see Table 1). Only in a later stage we lifted this constraint. For one case (Event #4), we noted a considerable improvement in the fit for a solution with a field above the clouds. In this case, we also added a fourth layer. For the other events, adding an additional layer did not improve the fit sufficiently to keep it or the fit was already of such a quality that there was no need for trying a more complex configuration. As discussed in section 3, the charge layers are expected at the heights *h*_*i*_. As mentioned earlier, we only consider the field **E**_⊥_, which is perpendicular to the shower axis as **E**_‖_ has very little effect on the radio emission in the frequency range from 30–80 MHz (Schellart et al., 2015; Trinh et al., 2016). In the actual shower simulations, we therefore set **E**_‖_=0 in this work. For a vertical shower, the field **E**_⊥_ is horizontal. For realistic inclined showers, **E**_⊥_ contains both horizontal and vertical components.

As argued in Trinh et al. (2016), the drift velocity and thus the amplitude of the radio signal is proportional to the strength of the perpendicular electric field **E**_⊥_ when it is small. When the electric field becomes larger than about 50 kV/m, the increased drift velocity results in a decreased longitudinal velocity. Thus, these particles trail further behind the shower front and their radiation loses coherency in the LOFAR frequency range. This results in a less than linear increase of the radio amplitude for fields exceeding about 50 kV/m. At a strength of 100 kV/m, the radio emission saturates in the sense that it is no longer dependent on the strength of the field. In the fits, we thus constrain the field not to exceed this value (by much).

The position of the shower core is essential for accurate fitting. In principle, the core position can be determined from the particle measured by the LORA array. However, analysis of fair‐weather data suggests that the actual core position, as determined from the radio data, can easily be displaced by as much as 50 m from the core position extracted from the LORA data. Therefore, in fair‐weather events, the core position is found by performing a combined fit for both radio and particle data (Buitink et al., 2014). For thunderstorm events, we follow the same procedure.

The fitting procedure for thunderstorm events is performed in two steps. In the first step, we use MGMR3D to optimize the parameters in the electric field profile to obtain a best fit for the Stokes parameters. Since the current is the product of the drift velocity, mainly determined by the strength of the electric fields, and particle number, mainly determined by *X*_max_, the strength of the electric fields can compensate a change in *X*_max_ to a certain extent. Thus, *X*_max_ and the electric field strengths are not independent. For this reason, we fit the parameters in the electric field profile while keeping *X*_max_ fixed. By using a Levenberg‐Marquardt minimization procedure, which is based on a steepest descent method, we optimize the parameters of the electric field configuration by minimizing

(5) $$\chi_{3D}^{2}=\underset{\text{antenna}\;k}{\sum }\overset{Q,U,V}{\underset{S=I}{\sum }}{\left(\frac{S_{k}-f_{r}^{3D}S_{k,\text{cal}}}{\sigma_{k}^{S}}\right)}^{2}\;.$$

Here, *S*_*k*_ denotes the measured Stokes parameter (calculated from a 55‐ns window around the peak) for antenna number *k* with an uncertainty $\sigma_{k}^{S}$, and *S*_*k*,cal_ is the calculated value for the Stokes parameter. $f_{r}^{3D}$ is an overall scaling factor for the radio intensity. The absolute intensity, related to the energy of the shower, will be determined in the following step in conjunction with the particle distribution.

For each event, three values of *X*_max_ are chosen that seem plausible given the typical fluctuations one observes in *X*_max_. For each value of *X*_max_, we generally fit, using the MGMR3D code, the nine parameters of the field configuration (three heights, magnitude, and angle), two parameters for the core position, and the energy of the cosmic ray (acting as a normalization factor). In this fitting, the height of the upper layer, *h*_1_, is initially set at the height of the echo top. When also good results can be obtained with two layers, such a solution is preferred. When the chi‐square exceeds 2, we have explored a field configuration with four layers. This has led for Events #1 and #4 to include a field well above the cloud top height as inferred from the echo top. The obtained results will be discussed in detail in section 2.4. For each value of *X*_max_, the best fits obtained in MGMR3D are called Calculation (Cal.) I, Cal. II, and Cal. III (see the supporting information).

In the second step, we perform CoREAS simulations using the parameters of the atmospheric electric fields as determined in the MGMR3D calculations Cal. I–III. In CoREAS calculations, the value of *X*_max_ cannot be set since it is determined by the stochastic development of the air shower. For this reason, we perform 20 calculations with different seeds for each of the electric field configuration as determined from the MGMR3D fits where we keep the calculations that have the best $\chi_{C}^{2}$ value (see equation (6)). This value of *X*_max_ is, not surprisingly, very close to the one that was used in the MGMR3D calculation (differences are less than 15 g/cm^2^). The CoREAS simulations corresponding to Cal. I–III are called Simulation (Sim.) I, Sim. II, and Sim. III. For the CoREAS simulations, $\chi_{C}^{2}$ values are calculated for the combination of Stokes parameters and the particle distribution

(6) $$\chi_{C}^{2}=\underset{\text{antenna}\;k}{\sum }\overset{Q,U,V}{\underset{S=I}{\sum }}{\left(\frac{S_{k}-f_{r}S_{k,\text{sim}}}{\sigma_{k}^{S}}\right)}^{2}+\underset{\text{LORA detector}\;j}{\sum }{\left(\frac{D_{j}-f_{p}D_{j,\text{sim}}}{\sigma_{j}}\right)}^{2}\;,$$

where *S*_*k*,sim_ is the simulated Stokes parameter, *D*_*j*_ is the deposited energy measured by a LORA detector with an uncertainty *σ*_*j*_, and *D*_*j*,sim_ is the simulated deposited energy that is converted from the CORSIKA particle output by using a GEANT4 (Agostinelli et al., 2003) simulation of the LORA detectors. In equation (6), two scaling factors are introduced, the scaling factor for particle energy, *f*_*p*_, and the scaling factor for the power, *f*_*r*_, determined to minimize the chi‐square.

Since the number of particles on the ground is, to a good approximation, proportional to the energy of the shower (when keeping *X*_max_ fixed), the energy in the simulation, *E*_sim_, is chosen such that the scaling factor for particles, *f*_*p*_, is unity. The scaling factor for the radio power, *f*_*r*_, obtained from the CoREAS simulations, remains as a parameter. The units are chosen such that for fair‐weather events, *f*_*r*_=1. *f*_*r*_ is linearly proportional to *E*_⊥_ if the fields are smaller than 50 kV/m. In this regime, the scaling factor can thus be used to determine the strength of the fields. In most realistic cases, however, one of the fields in the fit is stronger than 50 kV/m, and this proportionality cannot be used any more. Thus, *f*_*r*_ is important for determining the quality of the fit.

The fit parameters and the reduced *χ* ^2^ values obtained from both MGMR3D and CoREAS are given for each event in separate tables in the supporting information. The best or preferred fit, marked with an asterisk in the tables given in the supporting information, is chosen based on the reduced $\chi_{C}^{2}$ (see equation (6)) obtained by CoREAS simulations in combination with the extracted normalization factor *f*_*r*_ for the radio intensity. For example, as shown in Table S4 for Event #4, all three CoREAS simulations give almost the same reduced *χ* ^2^ value. However, since the value of the radio scaling factor *f*_*r*_ in Sim. I is the smallest, this calculation is preferred. It should be noted that the extracted structure of the fields does not depend on the value of *X*_max_.

Reversing the direction of the induced current in all of the layers, which, in the absence of the Earth magnetic field, is the same as changing the direction of the atmospheric electric fields, changes the polarity of the radiated field measured at the ground, but not the values of the Stokes parameters. This is because the Stokes parameters are based on measured power values and indicate the orientation of the polarization vector, but not the direction in which the vector is pointing. For every measurement, there are thus two solutions with the same chi‐square differing only in the orientation of the induced current in the air shower. The resulting ambiguity is resolved by taking the solution that gives the correct polarity of the observed waveforms. As an example, Figure 1 shows the comparison between calculated and measured pulses at a distance of 125 m from the shower axis for Event #8. As shown in the figure, the $v\times \left(v\times B\right)$ component of the pulse between MGMR3D and data is similar. The **v**×**B** component has some differences, but it is dominated by noise. It has been confirmed that inverting the current profile changes the overall sign, making the calculation inconsistent with the data.

### 2.4 Electric Field Determination

As an example of the rich structures that are measured in the radio footprints, we show Figures 2 and 3. The intensity and circular polarization patterns observed in Event #6 (see red points in Figure 2) resemble those of a fair‐weather event; however, the linear polarization is rather different. Instead of unity, we find here *Q*/I≈0, which means that the polarization vector is making a ±45° angle with the **v**×**B** direction. Since *U*/*I*≈−1, the angle is actually −45°. The fact that the circular polarization is small, *V*/*I*≈0 implies that the fields in the different layers are mostly oriented along the same direction as there should not be a net rotation angle in the fields when going from one layer to the next. The blue points in Figure 2 show the results of a CoREAS calculation (Huege et al., 2012) using the three‐layered structure for the atmospheric electric field as specified in Table 1. For the true force acting on the electrons and positrons, the geomagnetic force has to be added, resulting is a net force in the −45° plane. The Stokes parameters for Event #7 occurring only 10 min later (Figure 3) show a completely different structure. Here, we see clear ring structure in intensity with a diameter of close to 200 m, indicative of a strong destructive interference between different layers. The strong circular polarization near the core is evidence that the field orientations in the different layers have a definite twist. From the values of the atmospheric electric field given in Table 1, one can see that this is indeed the case.

For Event #1, a reasonable fit could be obtained with MGMR3D; however, the corresponding CoREAS calculation differs greatly from the semi‐analytic result, much to our surprise. The reason for this discrepancy is not understood, but is most likely due to the fact that for the present solution, there is a strong destructive interference between the emission from the topmost layer and the layer below that. A destructive interference has the tendency to magnify small differences between the two codes. Another reason could lie in the fact that this event has a large zenith angle, of about 40°. However, because of the observed discrepancy, we cannot trust the structure for the atmospheric fields that have been obtained for a fit using MGMR3D, and for this reason, we drop Event #1 from future considerations.

### 2.5 Summary

The analysis of the radio footprint of cosmic rays, CRCT, is a tomographic procedure to determine atmospheric electric fields. The advantages are that it is nonintrusive and it is a freely available side product of the ongoing program of measuring cosmic rays at LOFAR. The downside of the approach is that it requires a parameterized vertical electric field profile that is necessarily simplified. In addition, we can determine the component perpendicular to the cosmic‐ray direction only.

In this reporting period, the number of good‐quality thunderstorm events was only 11, since this covers the initial period of cosmic‐ray observations at LOFAR, the uptime was limited. In addition, there are relatively few thunderstorms in the Netherlands. The important measure is, however, the time between good events going through the same cloud (see section 3.4). The present sample of 11 events already contains two groups of three events that occur within half an hour and thus potentially offer the possibility to reconstruct all three components of the electric fields. In the future, this number will increase as the area of the LORA particle counters, needed for triggering, is being increased by a factor 2, thus strongly increasing the chance for multiple rays in a short time span. In addition, there is the possibility of decreasing the threshold in the particle detectors to increase the count rate.

## 3 Discussion

The atmospheric electric fields, as we have been able to determine for 10 out of the 11 good‐quality showers where the observed radio footprint strongly differs from that expected for fair‐weather showers, are given in Table 1. Listed are the UTC time, the zenith angle, *θ*, and the azimuth angle, *ϕ* (see also Figure 9), and the extracted atmospheric field configuration, parameterized as discussed in section 2.3. The component of the field **E**_⊥_, perpendicular the direction of the cosmic ray (given by **v**), is specified as the magnitude of the vector, *E*, and its angle *α* w.r.t. the direction of the magnetic Lorentz force, *e*_**v**×**B**_. **E**_⊥_ is also specified in terms of its true horizontal component, *E*_**v**×**z**_, where **z** is the vertical, and $E_{v\times \left(v\times z\right)}$, which is slanted, that is, a mix of horizontal and vertical. It should be noted that some of the extracted electric fields are vanishingly small. One example is that for the lowest layer of Event #3.

From Table 1, it is seen that for most events, we could get satisfactory results when restricting the top layer to (approximately) that of the echo top height. Notable exceptions are the two winter Events #2 and #3 and especially Event #4 of April 2012. For all three cases, the top height is rather low, and it could be that there have been thin high‐lying clouds that escaped detection. For the other Winter Event #9, no evidence is found for such a high‐lying charge layer. For many of the summer events, the upper charge layer lies well below the echo top height. It should be noted that for Event #4 (like for Event #1), the CoREAS results differ more from the best fit MGMR3D calculation than for the other events, and thus, the field above the echo top might be an artifact due to a not optimal parameterization of the field structure for this case. A qualitative discussion of the uncertainties of the extracted field is give in section 3.4.

### 3.1 Charge Layer Interpretation

In our analysis, we determine the components of the electric fields that are perpendicular to the direction of the cosmic‐ray shower and the heights at which these components change. The interpretation of these field changes in terms of the positions of different charge layers is however ambiguous.

One possibility is that the air shower passes through a horizontal charge layer. At this position, the vertical component of the field will change keeping the horizontal component unaffected. Since the shower is generally at an angle with the vertical, also the component perpendicular to the cosmic ray will change. This even more so when the charge layer is not really horizontal. In this picture, the charge layers are located at the heights where the fields change.

Another possibility is that the cosmic ray passes at some distance from a horizontal charge layer. In this case, the horizontal field will be large at the height of this charge layer and also large but in an opposite direction at the height of the following layer that will have an opposite charge. The horizontal component will thus change right in between the charge layers. The charge layers will thus be located in between the heights where the fields change. Charge layers in this respect are anything that affects the electric field, including charged precipitation (Marshall & Winn, 1982) and that corona discharge on sharp points on the ground (such as fences and trees), which is only significant at low altitude (Standler & Winn, 1979)

**Figure 4:**
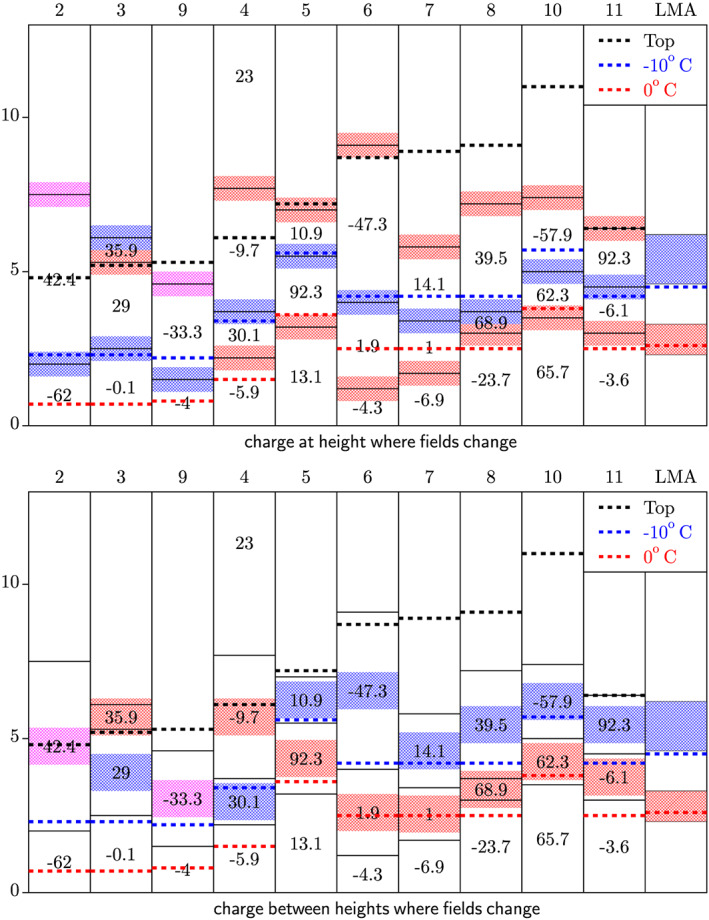
The estimated positions of the charge layers is shown, based on the extracted field as given in Table 1. The left panel is based on the interpretation that the charge layers are located at the heights where the fields change, the right one assumes instead that the horizontal component changes at an height in between charge layers. Suggested positive charge is indicated in red, while suggested negative charge is given in blue. When there is no apparent suggestion, magenta is taken. Also indicated are the heights of the 0 and −10 °C isotherms as well as the echo top heights. The numbers give the values of the *E*_**v**×**z**_ component (in kV/m) of the field, which is purely horizontal. The column “LMA” gives the charge layers as determined from the LMA observations given in Figure 5.

**Figure 5:**
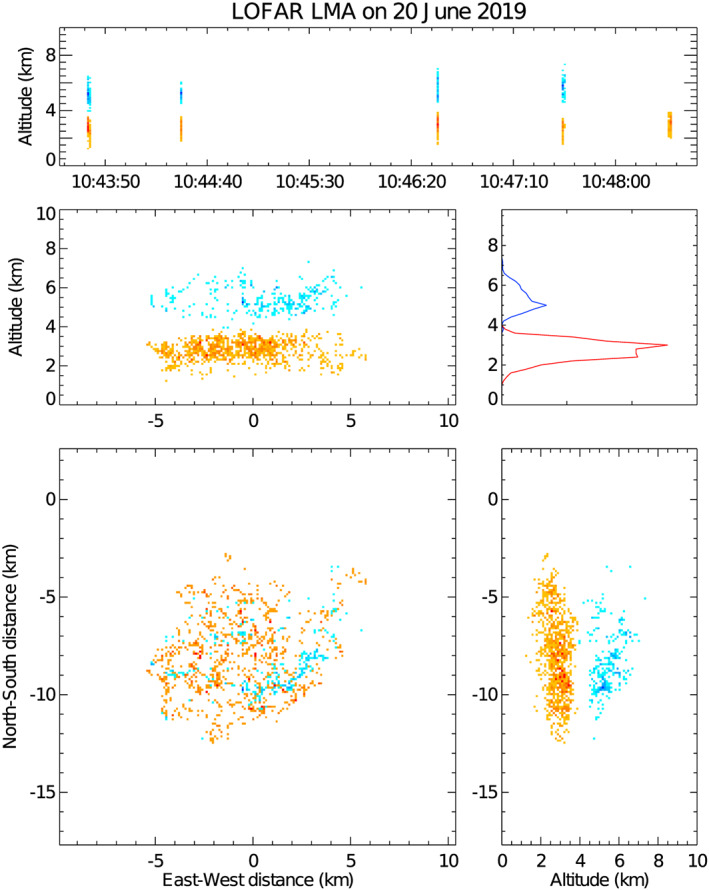
LMA data from a few lightning flashes recorded near the LOFAR core (at *X*=0 and *Y*=0) on 20 June 2019. The orange points show the location of negative lightning leaders propagating through positive cloud charge. The blue points show the location of positive lightning leaders propagating through negative cloud charge.

In Figure 4, the two interpretations for the charge layer structure are set side by side together with the isotherms as determined from GDAS observations. The first few events are detected in winter where the 0 °C isotherm lies at rather low heights where our method loses sensitivity. In general, one expects the main negative‐charge layer between −10 and −20 °C (Krehbiel, 1986; Mansell et al., 2005), which is supported by laboratory experiments (see Takahashi & Miyawaki, 2002). This seems to be more consistent with the second interpretation for the position of the charge layers. This is supported by LMA observations, as shown in Figure 5, of a flash that occurred near the LOFAR core, recorded on 20 June 2019. Using a standard LMA charge analysis, this flash shows that there is a negative‐charge layer at 2.3–3.3 km and a positive‐charge layer at 4.7‐ to 6.3‐km altitude. Note that this style of analysis can miss weaker charge regions, so based on the traditional three‐layer thunderstorm charge model, we expect another positive‐charge layer around 8‐km altitude.

On the other hand, in Florida thunderstorm observations (Pilkey et al., 2014), it was determined that in some cases, the charge layer was horizontal and in some others strongly inclined. For inclined charge layers, one expects the horizontal component to change at the level of the charge layer. Inclined layers are also seen in the thunderstorm charge structure simulations by Mansell et al. (2005). One should however keep in mind that Pilkey et al. (2014) have determined that the main negative layer lies rather at the −10 °C isotherm. Here, one should keep in mind that the position of the isotherm was determined from some averaged atmospheric measurements in the general area, comparable to our GDAS data, and not really based on the actual temperature structure of the thunderstorm. Another reason for placing the charge layers at the heights where the fields change is that for those clouds where all three components of the field can be reconstructed, as discussed in section 3.4, the analysis results in rather strong vertical components of the electric field changing sign at the same heights where the perpendicular components change. Since the fields determined by LOFAR should be seen as some average fields, this favors the first interpretation, that is, placing the charge layers at heights where the fields change.

### 3.2 Meteorological Conditions During Events

There were four thunderstorm events (#1, #2, #3, and #9) measured during the winter, six events (#5, #6, #7, #8, #10, and #11) during the summer, and one event (#4) in April. In particular, there were three events recorded within 12 min in a winter night and three events measured within 36 min in a summer day. This allows a comparative analysis that will be discussed in section 3.4.

In order to be able to interpret the heights of the charge layers obtained in the previous section, we discuss the meteorological conditions at (or near) the LOFAR Superterp, based on data from the Météorage lightning detection network as well as radar data. Because the maximum zenith angle of the different events is 39.4° (see Table 1), and we can assume that charge layers occur no higher than 15 km, we are interested in meteorological conditions within approximately 10 km of the Superterp location. The presence of lightning is an accurate indicator of the existence of strong electric fields (resulting from vertically separated layers of charge). For Events #6–8, #10, and #11, there was lightning directly overhead (see the supporting information). For Event #9, there was lightning in the vicinity but farther away than 10 km from the LOFAR Superterp. This indicates that the meteorological conditions were favorable for cloud electrification to occur. There was no lightning activity in the vicinity for Events #2, #3, #4, and #5, but still, we observed a considerable electric field for these events. For all events, the atmospheric conditions are discussed in the supporting information; however, here, we elaborate on the conditions for the summer and winter events that occur in a short time span in sections 3.2.2 and 3.2.1 respectively as these are of particular interest.

**Figure 6:**
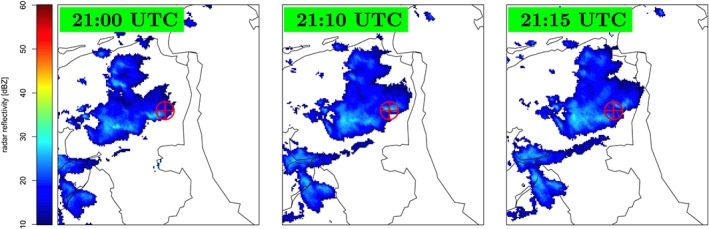
Radar reflectivity in dBz as determined for different UTC times on 14 December 2011. The red ⊕ marks the location of the LOFAR “Superterp.” There were three events measured on this day. Event #1 was measured at 21:02:27 UTC, Event #2 at 21:10:01 UTC, and Event #3 at 21:14:34 UTC. Each picture shows an area of about 160×180 km^2^. Data from ADAGUC (2018).

It is known that cloud electrification occurs in regions with large updrafts and when there is a sufficient amount of precipitation particles present (e.g.,  MacGorman & Rust, 1998). This means that conditions required for charge separation (Mattos et al., 2017) have specific signatures in radar data, with high low‐level reflectivity values (e.g.,  Dixon & Wiener, 1993), and high echo tops (e.g.,  Ushio et al., 2001). Radar reflectivity at 1,500  m altitude and the maximum height at which radar reflectivity is at least 7 dBZ (i.e., echo top heights) derived from the operational weather radar network of the Netherlands is used here to study whether the conditions for cloud electrification are met. Furthermore, echo top heights can also be linked to the height of the charge layers derived for the different events. The figures of radar reflectivity such as Figure 6 show that radar reflectivity values exceed 35 dBZ for all summer events (including #4). Note that for these events, the 0° isotherm is well below the 1,500 m level (at which radar data are displayed; see Table 1), resulting in a high fraction of ice in the radar measurement volume, leading to lower reflectivity values. Echo tops are at least ∼5 km high for all of the events, with a maximum echo top at 11 km for Event #10. This indicates that for all of the events, the meteorological conditions were favorable for cloud electrification to occur at or very close to the LOFAR Superterp.

#### 3.2.1 Winter Events

Interesting to note is that the lowest height where the fields change for the three analyzed winter events (#2, #3, and #9) appear to lie close to the −10 °C isotherm. The reason for this is most probably that the 0 °C isotherm lies too low to have a measurable effect on the radio footprint of the cosmic‐ray showers and the associated positive‐charge layer is stretched to the ground. Note that in extracting the electric field structure, the heights of the isotherms were not taken into account.

For the three events (#1, #2, and #3) detected on 14 December 2011, there was no lightning activity detected in the vicinity of the Superterp. Radar reflectivity measurements (Figure 6) show that at the time of the air shower detections, a rather large cloud was passing over the Superterp with several active cells passing right over the Superterp. All three events were detected within a time span of 12 min, however, and most unfortunately, the reconstruction of the radio footprint of Event #1 was not successful.

#### 3.2.2 Summer Events

**Figure 7:**
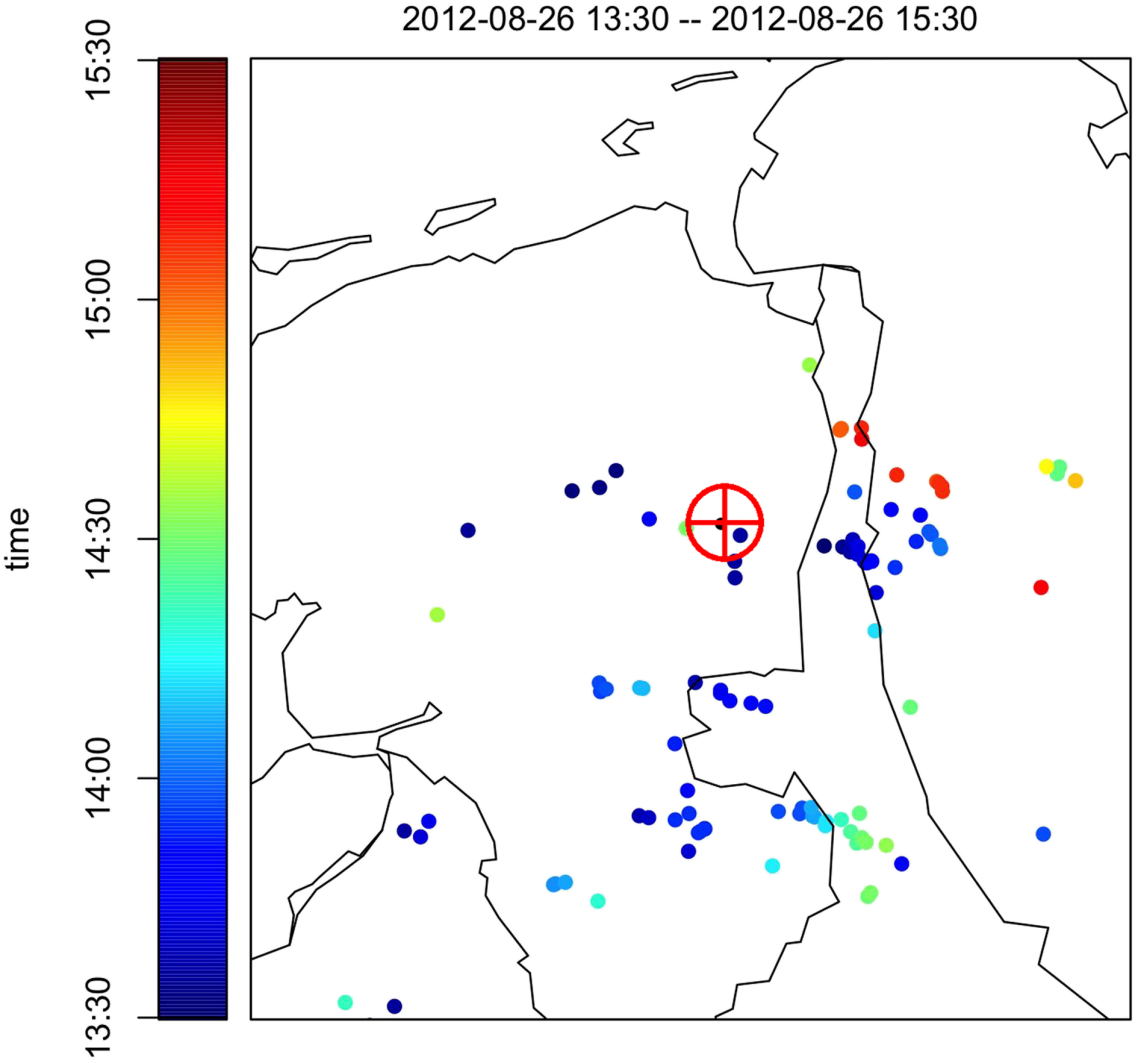
Lightning discharges on 26 August 2012 between 13:30 and 15:30 UTC as indicated by the color of the points. The red ⊕ gives the location of LOFAR “Superterp.” There were three events measured on this day. Event #6 was measured at 13:52:23 UTC, Event #7 at 14:02:56 UTC, and Event #8 at 14:28:19 UTC. Data from KNMI (2018).

**Figure 8:**
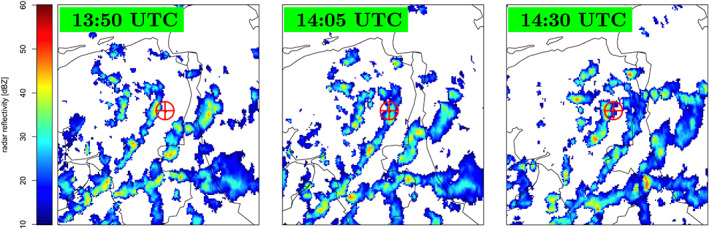
Same as Figure 6 for 26 August 2012 when there were three events measured. Event #6 was measured at 13:52:23 UTC, Event #7 at 14:02:56 UTC, and Event #8 at 14:28:19 UTC.

Events #6, #7, and #8 are special because these events are measured in a time span of less than 40 min on 26 August 2012 and will be discussed in more detail in section 3.4. During the time of detection of these events, there was lightning activity observed in close vicinity of the Superterp by the Météorage lightning detection network (see Figure 7). As can be seen from the figure, the lightning activity was spread over a larger area without a clear localization. All three events occurred within a time span of 36 min; however, the radar reflectivity images (Figure 8) clearly show that while Events #6 and #7 passed through opposite sides of the same cloud, Event #8 passed through a different one. From this picture, it is also clear that the thunderclouds are small as compared to tropical storms. Comparing the radar reflectivity images at sequential times, it can be seen that the clouds were moving rather fast from west to east. The same is also deduced from the time progression of the lightning activity.

### 3.3 Horizontal Electric Fields

**Figure 9:**
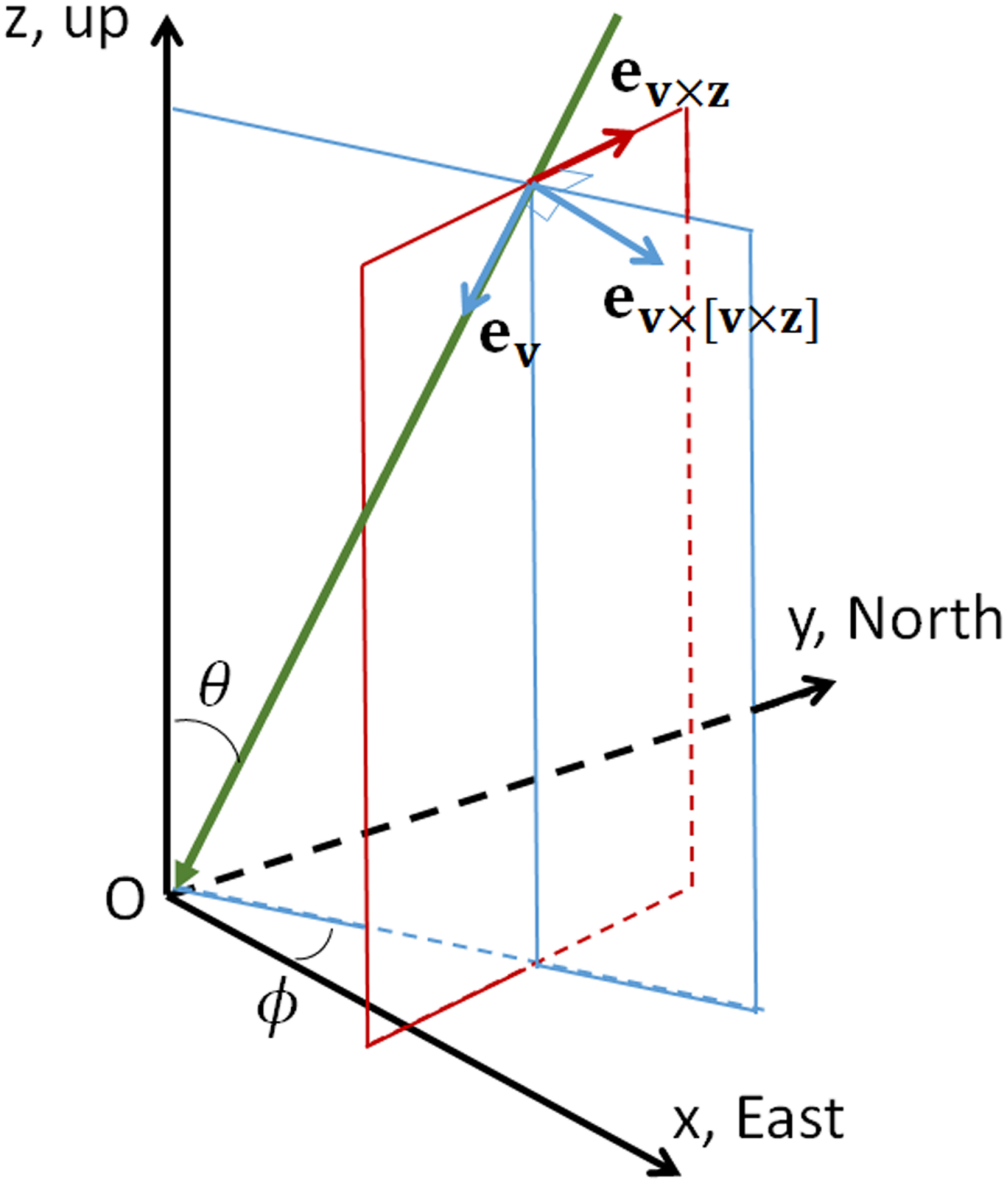
Direction of the air shower (thick blue line), $e_{v}$, and the two orthogonal unit vectors *e*_**v**×**z**_ and $e_{v\times \left(v\times z\right)}$ that are perpendicular to the shower. The electric field can be measured the directions *e*_**v**×**z**_ and $e_{v\times \left(v\times z\right)}$.

From our observations, we are able to determine the electric field component that is perpendicular to the shower axis, **E**_⊥_. Since generally the cosmic rays are not vertical, it is of interest to check what these observations imply for the horizontal component of the field. To obtain this, we decompose (Trinh et al., 2017) **E**_⊥_ into **E**_**v**×**z**_ and $E_{v\times \left(v\times z\right)}$ components of the field along the *e*_**v**×**z**_ and $e_{v\times \left(v\times z\right)}$ directions (see Table 1). Here, **v** denotes the direction of the shower and **z** is vertical (see Figure 9). *e*_**v**×**z**_ denotes a unit vector in the **v**×**z** direction and similar for $e_{v\times \left(v\times z\right)}$. **E**_**v**×**z**_ is purely horizontal as it is orthogonal to the vertical. This component together with the $e_{v\times \left(v\times z\right)}$ component, being perpendicular to both *e*_**v**×**z**_ and the shower, is thus a suitable basis for decomposing the electric fields determined in this work. It should be noted that $E_{v\times \left(v\times z\right)}$ has also a component in the vertical direction.

The components **E**_**v**×**z**_ and $E_{v\times \left(v\times z\right)}$, calculated for each event and all layers, are given in Table 1. We observe large horizontal electric fields in all 10 analyzed thunderstorm events with the exception of Events #6 and #7.

**Figure 10:**
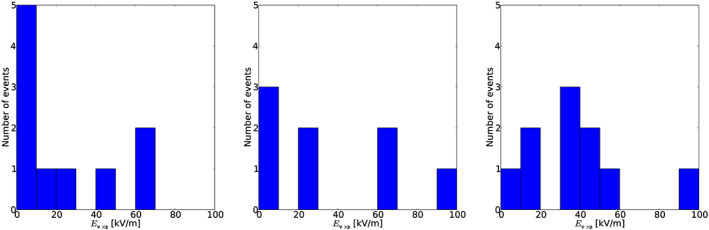
*E*_**v**×**z**_ distribution, left: bottom layer, middle: middle layer, and right: top layer.

It is interesting to analyze the distribution of the horizontal components. Figure 10 displays the magnitude of **E**_**v**×**z**_ for the different layers. The left panel of the figure shows that the horizontal electric fields between the bottom layer and the ground are small for all events except Events #2 and #10. Inside the thunderclouds, the fields are stronger as shown on the middle and right panels of the figure. This is as one would expect since the horizontal component of the field is due to the fact that the charge layers are not purely horizontal or the event occurred at the edge of the charged layer. Since the charges on the ground are influenced by the charges in the bottom layer of thunderclouds and Dutch ground is flat, the electric field between the ground and the bottom charge layer is most likely vertical.

The magnitude of the horizontal components is comparable to those found in rocket observations (Winn et al., 1974) and is larger than what is reported from airplane observations (Mo et al., 2002). As shown in the following section and in line with the airplane observation, the field below and above the main negative‐charge layer is mainly oriented along the vertical direction, however with sizable horizontal components.

### 3.4 Complete Direction Reconstruction of Electric Fields

As mentioned in the previous section, we can only determine the perpendicular component **E**_⊥_ since LOFAR is not sensitive to the parallel component **E**_‖_ of the electric field along the shower axis. However, there are two groups of events that were measured in a short time span (see Table 1). By applying tomography, where the results of different independent measurements are combined, one can—in principle—reconstruct the total electric field **E**. A basic assumption is that the field in the thundercloud does not change (much) over the time span and area where the showers of these events pass through the cloud. Marshall et al. (1995) show that in two summer thunderstorms in New Mexico, the charge densities inside the thunderclouds are similar over a time period of 15 min and over a distance of about 3 km.

To perform complete direction reconstruction, we consider two showers *i* and *j* that are close in time and space and are coming from the **v**_*i*_ and **v**_*j*_ directions, respectively. The perpendicular components of the electric fields determined from these two events are $E_{⊥i}$ and $E_{⊥j}$. Assuming that the total fields **E** in the thundercloud where the showers of these two events pass through are the same, we thus obtain

(7) $$E_{⊥i}+E_{\|i}e_{v_{i}}=E=E_{⊥j}+E_{\|j}e_{v_{j}}\;,$$

where *E*_*i*‖_ and *E*_*j*‖_ are the magnitude of the parallel components of the electric fields in two events that cannot be determined directly from the LOFAR measurements. This system of vector equations can be written as scalar equations by taking the dot products of equation (7) with $e_{v_{i}\times v_{j}}$, $e_{v_{i}}$, and $e_{v_{j}}$, respectively, where *e* denotes a unit vector in a certain direction. One thus obtains

(8) $$E_{⊥i}\cdot (e_{v_{i}\times v_{j}})=E_{⊥j}\cdot (e_{v_{i}\times v_{j}})\;,$$

and

(9) $$\begin{array}{ccc} E_{\|i} & =\frac{E_{⊥j}\cdot e_{v_{i}}+(e_{v_{i}}\cdot e_{v_{j}})(E_{⊥i}\cdot e_{v_{j}})}{1-{\left(e_{v_{i}}\cdot e_{v_{j}}\right)}^{2}} & \\ E_{\|j} & =\frac{E_{⊥i}\cdot e_{v_{j}}+(e_{v_{i}}\cdot e_{v_{j}})(E_{⊥j}\cdot e_{v_{i}})}{1-{\left(e_{v_{i}}\cdot e_{v_{j}}\right)}^{2}}\;. \end{array}$$

The total electric field E is consistent for two events if equation (8) is obeyed. When this is the case, the *E*_‖*i*_ and *E*_‖*j*_ components can be calculated from equation (9) and the total field E can thus be derived.

**Figure 11:**
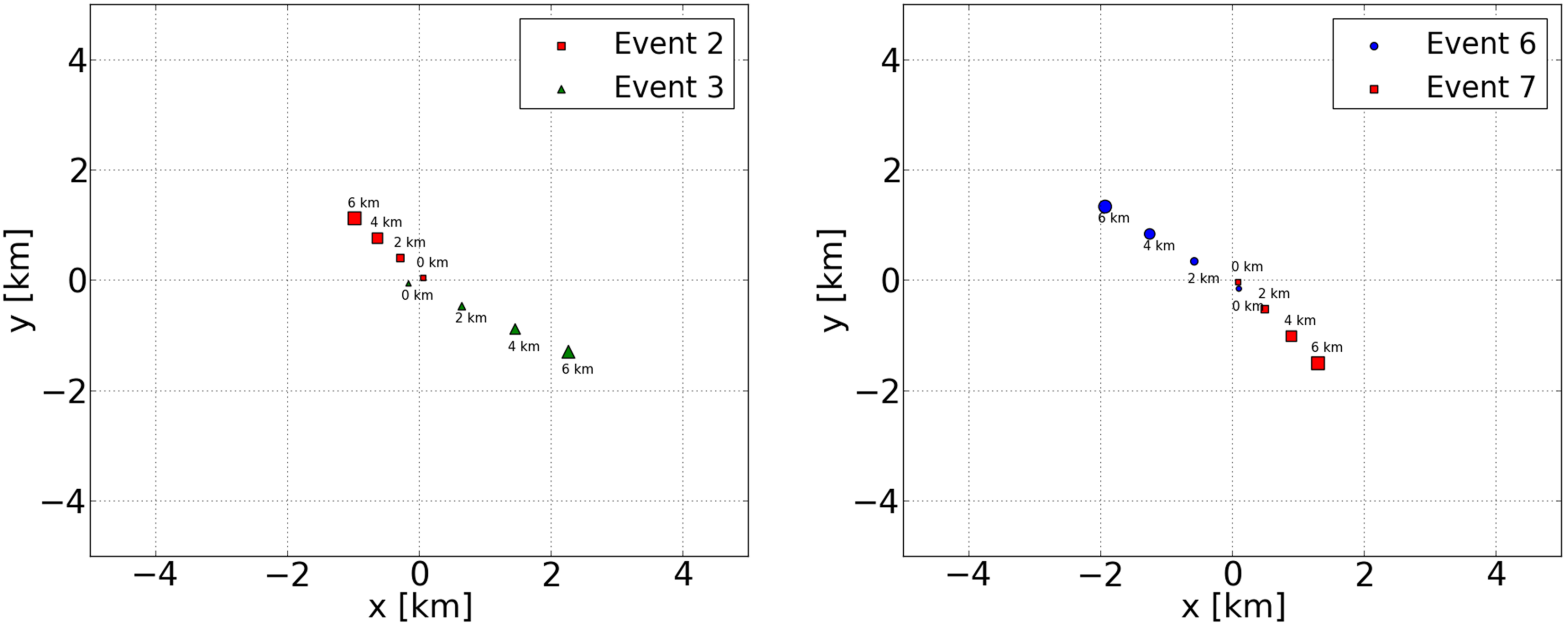
The core positions of the shower axis for Events #2 and #3 (left) and Events #6 and #7 (right) at different altitudes, as indicated.

Inspection of Figure 6 shows that Cosmic‐Ray Events #2 and #3 did pass through the same cloud. Inspection of Figure 8 shows that, of the three summer events that were measured in a small time window, Event #8 passed through a different cloud than Events #6 and #7, and for this reason, Event #8 should not be considered in the tomography analysis. It should be noted that there was no lightning activity observed during Events #2 and #3 while there was during Events #6 and #7. The core positions of these showers at different altitudes are shown in Figure 11. The figures show that at altitudes of more than 4 km, the shower cores tend to be several kilometers apart however along the wind direction, thus increasing the possibility that the showers test the same charge distribution.

To be able to judge if two showers move through a similar electric field configuration, as expressed by the criterium of equation (8), we need to have an estimate of the uncertainties in the extracted values of $E_{⊥}$. There are several factors that contribute to an error on the extracted fields, such as (a) measurement errors in the radio footprint that gives rise to a errors in the extracted fields from fitting the footprint; (b) ambiguity in the fit expressed by the fact that several values for *X*_max_ give almost equivalent solutions; (c) the approximations implicit to MGMR3D that are shown by the fact that its results differs from that by CoREAS; and (d) a relatively simple field configuration is used. Each of these factors is difficult to quantify. The error due to Point (a) is estimated to be negligible since the density of measurements is high enough such that the error in individual measurements can be averaged out. This can be seen clearly from Figures 2 and 3 where the fit shows systematic differences that are much larger than the error bars on the data points. The error due to Point (b) can be estimated on the basis of the variations we see in the fits for different values for *X*_max_ (see the supporting information). On the basis of this, we conclude that the results given in Table 2 are accurate to within 5 kV/m for the smaller field components increasing to 10 kV/m for components exceeding 50 kV/m. The errors due to approximations made in MGMR3D (Point (c)) are estimated to be small on the basis that the full CoREAS calculation shows very similar differences with the trends in the data as the MGMR3D calculations. We estimate this error to be of the same order as Point (b) or less. Most difficult is the estimate of the error introduced by taking a particular parameterization of the height dependence of the electric fields. It will not be possible to change the general structure of the fields, but, as again can be seen from Figure 2 and Figure 3, there is certainly room for improvement. This may be by smoothing the transitions from one layer to another or by taking different height dependencies for the *E*_**v**×**z**_ and $E_{v\times \left(v\times z\right)}$ components. This may give rise to fields that differ by 50 kV/m at certain heights, but averages over the ranges where the fields are taken constant in the present analysis should not change by more than 10 kV/m. To perform such an investigation falls outside the scope of this work. In total, the error margin is expected to be within 10 kV/m for the smaller field components increasing to 20 kV/m for components exceeding 50 kV/m.

**Table 2:** Checking the Consistency of Electric Fields Extracted From Events #2 and #3 and Events #6 and #7 (See equation (8))

Layer #*i*	Layer #*j*	$E_{i}\cdot (e_{v_{i}\times v_{j}})$	$E_{j}\cdot (e_{v_{i}\times v_{j}})$	$E_{i,z}$	$E_{j,z}$
Bottom #2	Bottom #3	50	−9	94	97
Top #2	Middle #3	−34	36	−139	−143
Top #2	Top #3	−34	31	8	2
Bottom #6	Bottom #7	4	−9	−15	−15
Middle #6	Middle #7	−2	13	113	114
Top #6	Top #7	43	10	−94	−95

*Note*. The first column lists the layers for the two cosmic‐ray events for which the extracted fields are correlated. The second and third columns list the extracted field in the perpendicular direction that is common to the two cosmic‐ray events. The vertical components of the fields, given in the last two columns, have been determined using equation (9). The quoted values are in kV/m.

Since the top heights of the bottom layers of Events #2 and of Event #3 differ by only 0.5 km (see Table 1), the consistency of electric fields for the bottom layers is checked. In Event #3, a three‐layered electric field is determined, while in Event #2, there are only two layers. One layer could be missing from Event #2 due to the fact that *E*_⊥_ in Event #2 hardly changes from 2 to 7.5 km. Therefore, we check in Table 2 the consistency of the electric field of the top layer of Event #2 with the middle layer and the top layer of Event #3. We see that the fields in none of the layers of these two events are consistent because the projections of $E_{⊥i}$ and $E_{⊥j}$ on the $E_{v_{i}\times v_{j}}$ direction differ by about 60 kV/m for all layers (see Table 2). The time difference between Events #2 and #3 is only 4 min, and the distance between two points where the showers of these two events pass through in the bottom layer is about 1.5 km. Therefore, it appears that in this cloud, the horizontal electric field, and thus the charge density, changes over a distance less than about 1.5 km and in a period less than 4 min. The radar reflectivity images in Figure 6 support this. At the time of the measurements, the clouds were moving at high speed from west to east, and at the time of Event #2, the active center of the cloud was overhead. In addition, the reflectivity images show cloud details at the kilometer scale.

In spite of the inconsistency of the perpendicular fields, we have calculated the vertical component of the fields. To our surprise, we find that for all three layers, the two ways of calculating yield very similar values. The vertical component is strong and positive for the bottom layer and strongly negative for the middle layer of Event #3. This corresponds to a large negative charge at the boundary at an height of 5.3 km, corresponding to the −10 °C isotherm, at the lower range where the main negative‐charge layer is expected. The inconsistency of the horizontal components can be explained by the fact that the two events occur at opposite sides of the cloud.

For the second group of Event #6 and Event #7, the top heights of the bottom and the middle layers of these two events are similar (see Table 1); thus, the consistency of electric fields of these layers is checked in Table 2. The top heights of the top layers of the two events are different by about 3 km. It could be that there is another electric field layer at about 5.8 km in Event #6 with a small *E*_⊥_, so we cannot determine it. As one can see from Table 2, the horizontal components of Events #6 and #7 are in reasonable agreement with each other for all layers, taking into account the error in determining these fields. Also for this case, we have extracted the vertical components of the field as shown in Table 2, and again, we see that these are very similar for the two possibilities. For these summer events, the −10 °C isotherm lies between the middle and the top layer and the signs of the vertical components are indicative of the main negative‐charge layer at this height.

## 4 Conclusion

In this work, we have used the radio footprint of cosmic ray‐induced air showers as measured at the LOFAR core to analyze 10 “thunderstorm” events measured from December 2011 to August 2014. The intensity patterns and the polarization signatures of these events, which are very different from the fair‐weather footprints, are reproduced reasonably well by assuming a three‐layer model of the atmospheric electric fields.

It is clear from lightning detection and radar data that all of the events occurred in conditions favorable for the occurrence of cloud electrification. For five events, #6, #7, #8, #10 and #11, there was lightning activity detected at the LOFAR core at the time when these events were measured (see Figure 7 and others in the supporting information). At the time of the other events, there were clouds with appreciable radar reflectivity. Our data thus have shown that in such clouds, the electric fields are large, approaching those observed in thunderstorm conditions.

For most of the events, the upper end of the electric field layers lies around the echo top heights. For some cases, mostly winter events with relatively low echo top heights, the altitude of the upper end of the field is well above the echo top. We also find for the two clouds where all components of the electric field could be reconstructed evidence for a negative‐charge layer near the −10 °C isotherm as determined from GDAS data. This finding agrees with Pilkey et al. (2014) where it was shown that, for Florida summer thunderstorms, the lower positive‐charge region tends to lie on the 0 °C isotherm and the negative‐charge region tends to lie near the −10 °C isotherm and sometimes even below.

For the lowest layer, the horizontal component of the fields is generally small; for the other layers, it is often large. For the cases where we could reconstruct the full field, we see that the horizontal component is strongly dependent on the location in the cloud, but the vertical components appear rather consistent.

In conclusion, we are able to determine from LOFAR data the heights of the charge layers for (near) thunderstorm clouds. In Hare et al. (2017, 2019), we have shown that LOFAR can also be used like a lightning mapping array to image the initial lightning discharge propagation in 3‐D. It will be most interesting to combine a measurement of the atmospheric fields with lightning imaging observations.

## Supporting information



Supporting Information S1
