# Supplementary material for: Determining Electric Fields in Thunderclouds With the Radiotelescope LOFAR
Source: J Geophys Res Atmos. 2020 Apr 22;125(8):e2019JD031433. doi: 10.1029/2019JD031433 (PMC7375151; doi:10.1029/2019JD031433)
Supplement: Supplementary file 1 — Supporting Information S1 [file JGRD-125-e2019JD031433-s001.pdf]

# Supporting Information for "Atmospheric electric fields measured by LOFAR"

T. N. G. Trinh[0,1], O. Scholten[1,2], S. Buitink[3,4], U. Ebert[5,6], B. M. Hare[1],

P.R. Krehbiel[14], H. Leijnse[7], A. Bonardi[4], A. Corstanje[4], H. Falcke[4,8,9,10], ,

T. Huege[12,3], J. R. Hörandel[3,4,8], G. K. Krampah[3], P. Mitra[3], K. Mulrey[3],

A. Nelles[11,13], H. Pandya[3], J. P. Rachen[3,4], L. Rossetto[4], C. Rutjes[5], S. ter

Veen[9], T. Winchen[3]

[0]Department of Physics, School of Education, Can Tho University Campus II, 3/2 Street, Ninh Kieu District, Can Tho City, Vietnam

[1]KVI-Center for Advanced Radiation Technology, University Groningen, P.O. Box 72, 9700 AB Groningen, The Netherlands

[2]Interuniversity Institute for High-Energy, Vrije Universiteit Brussel, Pleinlaan 2, 1050 Brussels, Belgium

[3]Astrophysical Institute, Vrije Universiteit Brussel, Pleinlaan 2, 1050 Brussels, Belgium

[4]Department of Astrophysics/IMAPP, Radboud University Nijmegen, P.O. Box 9010, 6500 GL Nijmegen, The Netherlands

[5]Center for Mathematics and Computer Science (CWI), PO Box 94079, 1090 GB Amsterdam, The Netherlands

[6]Department of Applied Physics, Eindhoven University of Technology (TU/e), PO Box 513, 5600 MB Eindhoven, The Netherlands

[7]KNMI, de Bilt

[8]NIKHEF, Science Park Amsterdam, 1098 XG Amsterdam, The Netherlands

[9]Netherlands Institute of Radio Astronomy (ASTRON), Postbus 2, 7990 AA Dwingeloo, The Netherlands

[10]Max-Planck-Institut für Radioastronomie, P.O. Box 20 24, Bonn, Germany

[11]Humboldt University of Berlin, Institute of Physics, Newtonstr. 15, 12489 Berlin, Germany

[12]Institut für Kernphysik, Karlsruhe Institute of Technology(KIT), P.O. Box 3640, 76021, Karlsruhe, Germany

[11]Erlangen Center for Astroparticle Physics, Friedrich-Alexander-Univeristät Erlangen-Nürnberg, Germany

[13]DESY, Platanenallee 6, 15738 Zeuthen, Germany

[14]Langmuir Laboratory for Atmospheric Research, Geophysical Research Center, New Mexico Institute of Mining and Technology,

Socorro, 87801, New Mexico, USA

**Contents of this file**

1. Outline of the procedure

2. General summary

3. Analysis data for each event #1 – #11

## 1. Outline of the procedure

This Supporting Information contains detailed results of the analysis for the 11 thunderstorm events that are the basis for the present analysis. We organize the events by day and there are plots of radar reflectivity (ADAGUC, 2018) in a wider area around the LOFAR 'Superterp'. It should be noted that the radar reflectivity measures precipitation which is a good proxy for the cloud coverage. If there was lighting activity near by or overhead the Superterp, we also show a plot of lightning discharges (KNMI, 2018). For each event, we include a table showing the results of the fitting process for each value of $X_{\text{max}}$. The preferred result is indicated with an asterisk attached to the simulation number in the table. The accompanying plot shows the results of the CoREAS calculation for the preferred parameters.

---

Corresponding author: T. N. G. Trinh, Department of Physics, School of Education, Can Tho University Campus II, 3/2 Street, Ninh Kieu District, Can Tho City, Vietnam (ttngia@ctu.edu.vn)

For all events the same procedure was followed where the parameters defining the structure of the atmospheric electric fields were fitted, using MGMR3D, in order to obtain the best agreement with the measured radio profile. In the fitting we optimized a chi-square criterium using the semi-analytic MGMR3D code for the calculation. This calculation was done for three different values for $X_{\rm max}$ since it was observed that there is a strong dependency in the parameters and thus it was not possible to keep $X_{\rm max}$ as an independent parameter. Once an atmospheric electric field configuration was obtained this was used as an input to a fully microscopic calculation, using CoREAS, to verify the results. In many cases the results of MGMR3D and CoREAS are very similar, but there were also some cases where they differ significantly. These cases were excluded from further analysis. Of the three calculation a preferred one was selected based on the agreement of the CoREAS calculation with the data and the value of the normalization constant, as explained in the main text. The preferred result is indicated with an asterisk attached to the simulation number in the table giving the fit parameters. For the preferred case a figure is shown giving the comparison of the results of the CoREAS calculation with the LOFAR data for the stokes parameters.

The determination of the position of the core of the air-shower is important for the analysis of the radio footprint. As a guidance the position as estimated from the particle-detector array (called LORA) is taken, in most cases this position was part of the chi-square search. The approximate shift in the core position is also noted.

### 1.1. General summary

All events show that over a wide range of $X_{\max}$ values, a reasonable agreement between MGMR3D and the data can be obtained. The extracted field configuration, in particular the boundaries of the different layers, is rather in-sensitive to the value of $X_{\max}$.

In events #1, #2, #4, #7, #8, and #10, the intensity pattern has a ring-like structure as can be seen from Fig. S1 which displays the intensity footprint of event #8. In the plots of the Stokes parameters (see the middle panels of Fig. S4, Fig. S5, Fig. S8, Fig. S15, Fig. S21), the ring-like structure is seen as a peak in the Stokes $I$ at a distance between 100 m to 250 m from the shower axis. The ring-like structure in intensity is due to a destructive interference between the radio emission from the upper and lower layers for these events since the field points almost in opposite directions in these layers. In addition, the radius of the ring in the intensity is strongly correlated to the height where the field is inverted. For smaller radii, the heights are smaller. For example, in event #2, the radius of the ring is 100 m and thus the field is inverted at 2.1 km (see Table S2) while in event #10, the radius of the ring is about 250 m and thus the field is inverted at 5.0 km (see Table S10).

In event #2, the amount of circular polarization (Stokes $V$) is very small (see the middle panel of Fig. S5). Thus, this event can be fitted by a two-layered electric field where the fields in the two layers are almost opposite to each other (see Table S2). In contrast, events #1, #7, #8, and #10 which also show the ring-like structure in the intensity have a large amount of circular polarization. Therefore, these events cannot be reconstructed well by a two-layered electric field structure. The electric field needs to have at least three layers (see Table S1, Table S7, Table S8, Table S10). A third layer is needed to

introduce the change in the orientation of the electric fields and thus the rotation of the transverse current which results in a large amount of circular polarization. In addition, a third layer also gives rise to the change in the linear polarization which causes a 'wavy' pattern. Fig. S2, as an example, shows the 'wavy' pattern of event #7 where the linear polarization rotates about 90° from small distances near the shower axis to large distances beyond 100 m from the shower axis.

Event #4 is an odd one since there is a large amount of circular polarization near the shower axis but the linear polarization is the same all antennas (see the middle panel of Fig. S8). Therefore, as shown in Table S4, the electric fields in the bottom and the middle layers are not fully opposite but they have an angle of about 150°.

In contrast to the events just discussed above, the intensity patterns in events #3, #6, #9, and #11 are similar to those in fair-weather events (see Fig. S6, Fig. S13, Fig. S18, Fig. S24). However, unlike for fair-weather events, with the exception of event #11, the signals are not polarized along the $\mathbf{v}\times\mathbf{B}$-direction because $Q/I$ is not equal to 1 (see Fig. S6, Fig. S13, Fig. S18). In events #3 as well as #6, since $U/I$ is about -1, the linear polarization makes an angle of about -45° with the $\mathbf{v}\times\mathbf{B}$-direction. For this reason, the electric fields in the layers where the current is large, i.e. the middle layer of event #3 and the top layer of event #6 (see Table S3 and Table S6), make an angle of about -55° with respect to the $\mathbf{v}\times\mathbf{B}$-direction. There is some amount of circular polarization in these two events but it is small. In event #9 which has been discussed in detail in (Trinh et al., 2017), the polarization footprint shows a 'wavy' pattern and there is a large amount of circular polarization, varying as a function of distance from the shower axis. Therefore,

the electric field in the middle and the bottom layer rotates 90° giving rise to the rotation of the linear polarization as well as the amount of circular polarization (see Table S9).

Event #11 is an odd case because not only the intensity but also the linear polarization looks like that of a fair-weather events at shorter distances to the shower axis, while at large distances $Q/I$ is much smaller than 1 (see the middle panel of Fig. S24). The main difference from a fair-weather event is, however, that the circular polarization is large and changes its handedness with distances which is caused by the rotation of the electric field orientation with height. Near the shower axis, the signal at the bottom layer arrives earlier than the signal from the other layers because the showers propagates with the speed of light while the signal moves at a reduced speed due to the finite refractivity of air. This gives rise to a large amount of circular polarization at small distances, $V/I$ = 0.4. At 150 m from the shower axis, the signal from the middle layer arrives earlier than the signal from the bottom layer, so $V/I$ = -0.4. Similarly, beyond 150 m, the signal from the top layer arrives before that in from the other layers, so the circular polarization continues to decrease at large distances.

### 1.2. December 14$^{th}$, 2011

For the three events detected on December 14$^{th}$, 2011, there was no lightning activity detected in the vicinity of the Superterp. The nearest lightning activity was detected at a distance of 200 km and we have thus not included a lightning map. Radar-reflectivity measurements, Fig. S3, show that at the time of the air-shower detections an active cell of a cloud was passing over the Superterp.

#### 1.2.1. Event #1

This event turned out to be the most difficult one to understand from our whole collection. Partly because of the lower intensity and thus relatively large error bars, partly because of the structure of the polarization. In addition the core location for this event is difficult to determine because of the low intensity of the radio signal and because the core appears to lie at the rim of the Superterp and is thus not surrounded by antennas. For a best fit in MGMR3D the core moved by about 70 m. In MGMR3D a good fit could be obtained for the three different values of $X_{\rm max}$ that does not differ much in structure. However, for each of these cases the results of the CoREAS calculation were showing considerable differences. This can be seen from the values of chi-square given in Table S1 by comparing $\chi^2_{3D}$ (for MGMR3D) with $\chi^2_C$ (for CoREAS). To give an example the results of the CoREAS calculation is compared with the data in Fig. S4. We do not understand the reasons for the major discrepancies between the semi-analytic and microscopic calculations for this event but it is probably due to strong destructive interference.

Because of the significant discrepancy between the data and the microscopic calculation we cannot be sure about the structure of the atmospheric fields and thus have decided to drop this event from further analysis.

#### 1.2.2. Event #2

The peak in the radio intensity for this event, see Fig. S5, is reached at distances of 100 m, indicative of a strong interference of the radiation from different layers. The radio intensity reaches a local maximum at the core telling that the radiation from the bottom layer is relatively large. Since $X_{\rm max}$ in Sim. I is small, the height where the number of particles reaches a maximum is in the top layer and thus the particle density becomes small in the bottom layer. For this reason, to have a large current at the bottom layer, the

electric field in this layer needs to be strong, as shown in Table S2. With increasing $X_{\mathrm{max}}$ the air shower penetrates deeper into the atmosphere and thus a smaller value for the electric field results in a similar emission strength for the bottom layer. The core position in the fit is shifted by mere 3 m from the position determined by the LORA data.

For this event the results of the MGMR3D calculation agree rather well with those from CoREAS for all three values of $X_{\mathrm{max}}$.

#### 1.2.3. Event #3

The determined field configurations for the different values for $X_{\mathrm{max}}$ do not differ greatly. In addition the results of the MGMR3D and the CoREAS calculation are close, although a discrepancy is seen for the intensity near the shower axis. We have selected Sim. III as the preferred one because of the slightly better, event hough the ratio $f_r$ is large.

### 1.3. April 26$^{\text{th}}$, 2012; Event #4

At the time of event #4 there was lightning activity detected at a distance of about 100 km however none within the vicinity of the LOFAR core. The radar-reflectivity data, Fig. S7, show that the shower passed through the edge of a rather extended cloud system with a very active core at about 20 km from the Superterp.

For this event the first two cases yielded a comparable agreement between CoREAS and MGMR3D as well as the structure of the electric fields, the values for the reduced $\chi^2$. The values for the norm factor $f_r$ as shown in Table S4 differ considerably. Initially we restricted the fields not to exceed the echo-top heights by much, however we noted that the chi-square improved by more than 1 unit when allowing for an electric field above the cloud. The maximum height of this layer is set at 15 km, any larger height would produce very similar results. To allow for this we have increased the number of layers to four for this event. We have selected calculation I as the preferred one. Since for this case the values for the electric fields are not very large one could have increased these and found a solution with a smaller value for $f_r$.

The observed ring-like structure in the radio intensity indicates that there is a considerable amount of destructive interference between the emissions from different layers. The rather large value of the circular polarization near the core is a sign that the fields must make a finite angle and are not completely pointing in opposite directions.

Particular for this event is that we needed to introduce a moderate electric field that extents well above the cloud height. We have tries to reproduce the structure without this field at large altitudes, but this resulted in a considerably worse value for the chi-square in the MGMR3D fits.

### 1.4. July 28$^{\text{th}}$, 2012; Event #5

For event #5 the only lightning activity is detected more than 12 hours after detecting the event. The radar-reflectivity data of Fig. S9 show that at the time of this event an extensive cloud was overhead that was not moving much. The shower passed through the edge of the cloud with an active core in close vicinity.

The radio intensity, Fig. S10, shows a clear maximum at the core position and one thus can deduce that there is little destructive interference in the radio footprint and the electric fields all point in basically the same direction, as is seen indeed from Table S5. The circular polarization shows a pattern that is similar to a fair-weather event. The direction of the linear polarization is however orthogonal to what one would expect for fair weather. All three fits show a very similar structure for the electric field configuration and similar values for the reduced $\chi^2$. Also for all three there is a good agreement between the CoREAS and the MGMR3D results. Since the norm factor for simulation I is smallest, combined with a good value for the reduced $\chi^2$ we have chosen this as the preferred result.

### 1.5. August 26$^{\text{th}}$, 2012

During the time of of detecting events #6, #7, and #8 there was some lightning activity observed by the Météorage lightning-detection network in the close vicinity of the Superterp, see Fig. S11. All three events occurred within a time span of 36 minutes, however the radar-reflectivity images, Fig. S12, clearly show that while events #6 and #7 passed through different sides of the same cloud, event #8 passed through a different one. The clouds were moving rather fast from west to east.

#### 1.5.1. Event #6

The three fits given in Table S6 show a three-layer field structure with similar strengths and orientations of the fields. The intensity and circular polarization patterns, see Fig. S13, are very reminiscent of those for fair weather circumstances however the linear polarization is *deleted: orthogonal* **new: at 45 degree**. The dip in the intensity near the core is an indication of some destructive interference between the emissions from different layers and thus a similar orientation of the electric field that is at a large angle to the of the geomagnetic force. Since for calculation I the agreement between the results of the CoREAS and the MGMR3D calculation is best we have selected this one as the preferred one. In addition the norm factor $f_r$ is best for this case.

#### 1.5.2. Event #7

The results shown in Table S7 shows that the extracted field configurations for the electric fields do not differ much for the three fits. In addition the CoREAS and the MGMR3D results agree well for all three cases. The radio-intensity pattern, see Fig. S14, shows a clear ring-structure with a rather large diameter. One thus obtains a field configuration where there is a strong destructive interference between the top layer and the lower two. The strong circular polarization is evidence for fields that are at a finite angle with respect to each other. We have selected simulation II as the preferred fit.

#### 1.5.3. Event #8

Also for event #8 one observes that the three different fits converge to very similar electric field configurations, see Table S8. Also for this event the results of the CoREAS and the MGMR3D calculations agree reasonably well for all three cases. For this event the core position was moved by 10 m from the original position determined from the LORA

data. The clear ring-like structure, see Fig. S15, in the intensity indicates a destructive interference between the contributions from different heights. Simulation III is selected as the preferred one.

### 1.6. December 30$^{\mathrm{th}}$, 2012; Event #9

At the time of detection of event #9 the nearest lightning activity was detected at a distance of 100 km, see Fig. S16. The radar-reflectivity images, Fig. S17, show that the clouds move fast from West to East and that at the shower must have passed right through the edge of an active cloud cell.

For this event one observes, see Fig. S18, an intensity pattern that closely resembles that of a fair weather event, however the polarization data differ completely. This results in rather stable orientations of the fields for the three fits, see Table S9, however the extracted strengths differ. The reason for this is that the height of $X_{\max}$ is right around $h_3$ and thus changes in $X_{\max}$ require a sizable compensation in the electric field to keep similar currents. Since this case could also be fitted well with a simple 2 layer structure, this is given preference. Simulation II is preferred based on the reduced $\chi^2$ as well as the ratio $f_r$. The core position was moved by 19 m. Even though the $\chi^2_C$ is worse than $\chi^2_{3D}$ one can see that the CoREAS fit is actually quite good since all the features in the data are reproduced. The $\chi^2_C$ is relatively poor because the data for this event has very small errors.

### 1.7. July 26$^{\mathrm{th}}$, 2013; Event #10

Considerable lightning activity was observed in the vicinity of the core at the time of detection of event #10, see Fig. S19. The radar-reflectivity data shown in Fig. S20

indicate much higher values than seen for most of the other cases as would be consistent with this being a real thundercloud with lightning activity. The shower passed very close to the most active region.

For this event the core position was moved by about 40 m from the original one determined from the LORA data. The three different fits for the electric-field configuration are very similar as shown in Table S10. For these cases the CoREAS results agree rather well with those from MGMR3D for simulations I and III, with larger discrepancies for simulation II. Combining this with the value for $f_r$ we have selected calculation I as the preferred one.

### 1.8. June 27$^{th}$, 2014; Event #11

Fig. S22 shows that there is lightning activity observed close to the core and at about the time of observing event #11. Comparing this with the radar-reflectivity data, Fig. S23, indicates that at the lime of recording this event the active cell was still only approaching the Superterp and still at a distance of about 50 km to the South-West. The rim of the thundercloud was just over the Superterp.

Also for this event one observes in Table S11 that the three different fits give very similar solutions for the electric field configuration. In addition for all three cases the CoREAS and MGMR3D results agree very well with each other. For this event the core of the shower had to be moved by about 60 m from the point suggested by the LORA detectors. We have seen signatures that for this event the reading of some LORA scintillator counters was affected by lightning. Such effects have been observed by the surface detector of the Pierre Auger Observatory (Colalillo, 2017). Simulation II is preferred because the ratio $f_r$ is close to unity.

## References

ADAGUC. (2018). *viewer version 3.0.4. for more information, please visit.* http://adaguc.knmi.nl/.

Colalillo, R. (2017). Peculiar lightning-related events observed by the surface detector of the Pierre Auger Observatory. In *The Pierre Auger Observatory: Contributions to the 35th International Cosmic Ray Conference (ICRC 2017)* (p. 138-145). Retrieved from http://inspirehep.net/record/1618429/files/1617990_138-145.pdf

KNMI. (2018). *KNMI lightning discharge data.* https://www.knmi.nl/nederland-nu/klimatologie/geografische-overzichten/onweer.

Trinh, T. N. G., et al. (2017). Thunderstorm electric fields probed by extensive air showers through their polarized radio emission. *Phys. Rev. D*, *95*, 083004. Retrieved from https://link.aps.org/doi/10.1103/PhysRevD.95.083004 doi: 10.1103/PhysRevD.95.083004

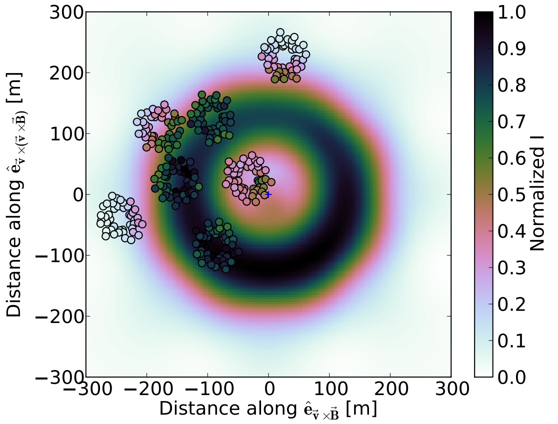


**Figure S1.** The intensity (Stokes $I$) footprint of event #8. The background color shows the simulated results while the coloring in the small circles represents the data. **new: The ring-like structure is clearly visible. When plotting the Stokes parameters v.s. distance to the shower axis, as done in Fig. S15, the ring structure shows as a peak in the intensity at at a distance of about 100 m. This structure is due to the interference of the emission from the upper layers with that from the lower layer (see table 1 of the main text).**

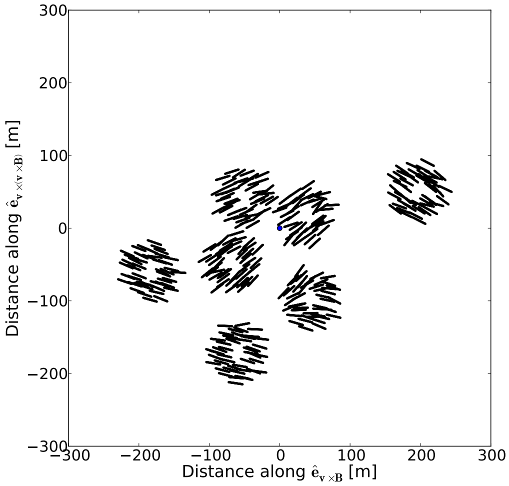


**Figure S2.** Linear polarization footprint of event #7 as measured with individual LOFAR LBAs (lines) in the shower plane. new: **The 'wavy' pattern for this case is due to currents that change in orientation at an height of about 3.4 km (see table 1 of the main text).**

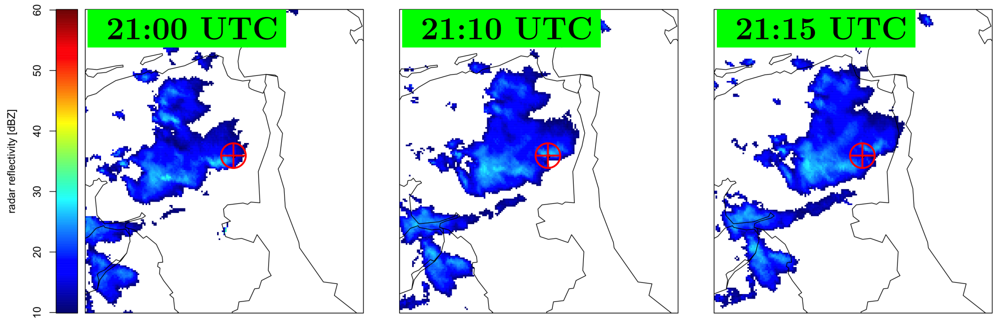


**Figure S3.** Radar reflectivity in dBz as determined for different UTC times on 14/12/2011. The red ⊕ marks the location of the LOFAR 'Superterp'. There were 3 events measured on this day. Event #1 was measured at 21:02:27 UTC, event #2 at 21:10:01 UTC, and event #3 at 21:14:34 UTC. new: **The nearest lightning activity was detected at a distance of 200 km.**

| Calculation | I | | | II | | | III | | |
|---|---|---|---|---|---|---|---|---|---|
| Energy (eV) | $8.4 \times 10^{16}$ | | | $8.5 \times 10^{16}$ | | | $3.3 \times 10^{16}$ | | |
| Layer | $h$ | $E$ | $\alpha$ | $h$ | $E$ | $\alpha$ | $h$ | $E$ | $\alpha$ |
| 0 | 11.3 | 21 | -183 | 15.0 | 22 | -180 | 15.0 | 33 | -188 |
| 1 | 5.5 | 60 | 114 | 6.2 | 40 | 125 | 5.6 | 39 | 117 |
| 2 | 4.8 | 107 | -72 | 5.0 | 106 | -68 | 4.6 | 110 | -71 |
| 3 | 3.1 | 66 | 92 | 3.3 | 62 | 102 | 3.1 | 43 | 103 |
| $X_{\max}$ (g/cm$^2$) | 560 | | | 634 | | | 743 | | |
| $X_{\max}$ (km) | 6.8 | | | 5.9 | | | 4.7 | | |
| $\chi^2_{3D}$ | 1.22 | | | 1.33 | | | 1.26 | | |
| $\chi^2_C$ | 16.5 | | | 6.1 | | | 14.3 | | |
| $f_r$ | 6 | | | 2 | | | 16 | | |

**Table S1.** The values of the fit parameters describing the structure of the atmospheric electric field as obtained from a chi-square fit using MGMR3D. Also given are the values for the chi-square for MGMR3D ($\chi^2_{3D}$) and that for CoREAS ($\chi^2_C$) using the same field and almost the same $X_{\max}$. The normalization factor for the intensity of the radio signal is given by $f_r$. This calculation is performed for event #1.

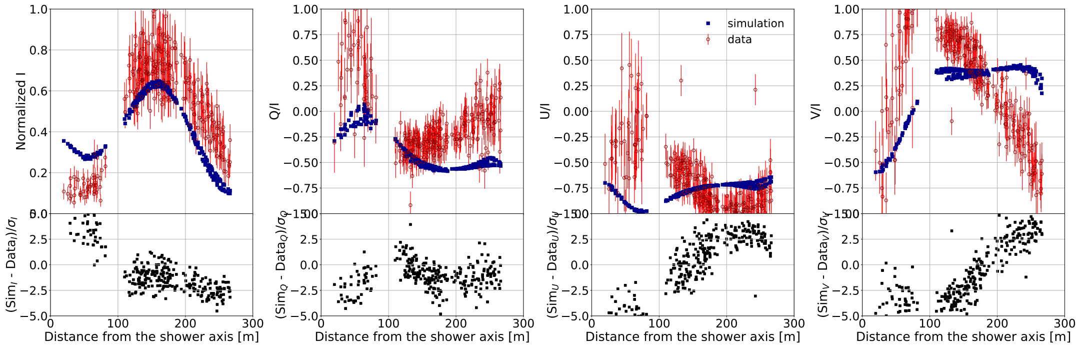


**Figure S4.** The results of Sim. II for normalized Stokes parameters (filled blue dots) are compared to LOFAR data (open red circles) for event #1. $\sigma$ denotes one standard deviation error.

| Calculation | I* | | | II | | | III | | |
|---|---|---|---|---|---|---|---|---|---|
| Energy (eV) | $3.2 \times 10^{16}$ | | | $3.1 \times 10^{16}$ | | | $1.9 \times 10^{16}$ | | |
| Layer | $h$ | $E$ | $\alpha$ | $h$ | $E$ | $\alpha$ | $h$ | $E$ | $\alpha$ |
| 1 | 7.5 | 53 | -171 | 5.8 | 92 | -171 | 5.1 | 89 | -171 |
| 2 | 2.0 | 82 | 13 | 2.1 | 78 | 11 | 2.0 | 55 | 12 |
| $X_{\max}$ (g/cm$^2$) | 595 | | | 645 | | | 690 | | |
| $X_{\max}$ (km) | 4.7 | | | 4.1 | | | 3.6 | | |
| $\chi^2_{3D}$ | 0.88 | | | 0.89 | | | 0.90 | | |
| $\chi^2_C$ | 1.32 | | | 1.45 | | | 1.45 | | |
| $f_r$ | 1 | | | 1 | | | 2 | | |

**Table S2.** Same as Table S1 but for event #2. **new: Since the intensity in the footprint shows a clear ring structure there should be a destructive interference of the radiation from different layers. To have comparable current in the two layers, the ratio of the electric field in the two layers compensates the ratio of the number of particles which changes with $X_{\max}$.**

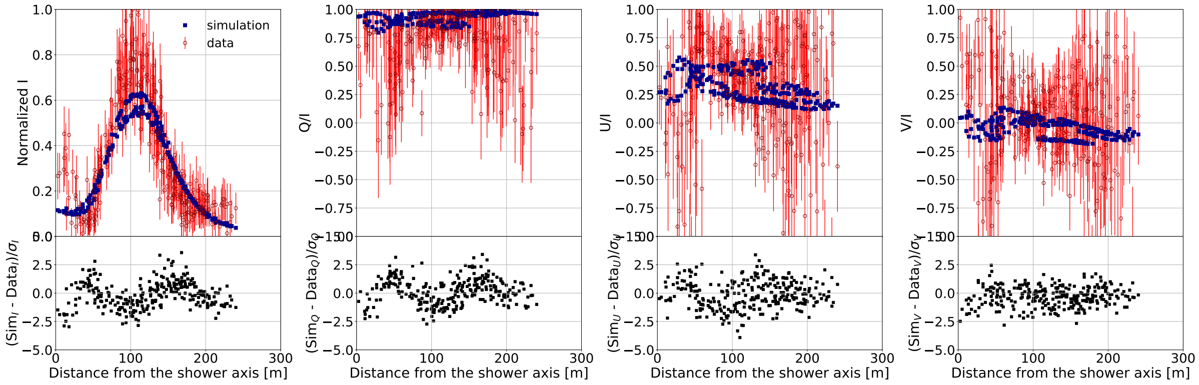


**Figure S5.** Same as Fig. S4 but for event #2 where simulation I is selected. **new: The ring-like structure is evident from the peak in the intensity at 100 m. The values for the other Stokes parameters are similar to those of a fair-weather event.**

| Calculation | I | | | II | | | III* | | |
|---|---|---|---|---|---|---|---|---|---|
| Energy (eV) | $3.5 \times 10^{16}$ | | | $3.2 \times 10^{16}$ | | | $3.1 \times 10^{16}$ | | |
| Layer | $h$ | $E$ | $\alpha$ | $h$ | $E$ | $\alpha$ | $h$ | $E$ | $\alpha$ |
| 1 | 6.6 | 46 | -151 | 6.2 | 46 | -156 | 6.1 | 52 | -170 |
| 2 | 5.6 | 56 | -60 | 5.5 | 47 | -62 | 5.3 | 63 | -62 |
| 3 | 2.4 | 3 | -4 | 2.5 | -1 | 172 | 2.5 | 0 | 7 |
| $X_{\max}$ (g/cm$^2$) | 620 | | | 670 | | | 720 | | |
| $X_{\max}$ (km) | 4.8 | | | 4.2 | | | 3.7 | | |
| $\chi^2_{3D}$ | 1.47 | | | 1.44 | | | 1.41 | | |
| $\chi^2_C$ | 2.74 | | | 2.62 | | | 2.75 | | |
| $f_r$ | 5 | | | 9 | | | 6 | | |

**Table S3.** Same as Table S1 but for event #3. new: **The field configurations are very similar for the three values of $X_{\max}$.**

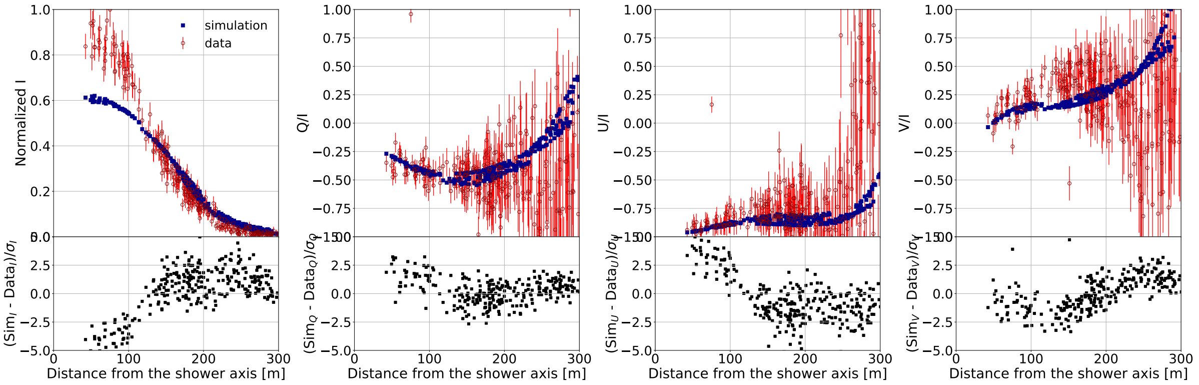


**Figure S6.** Same as Fig. S4 but for event #3 where simulation III is selected.

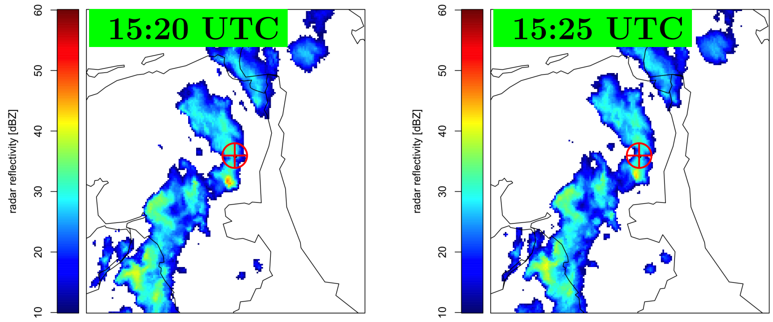


**Figure S7.** The red ⊕ marks the location of the LOFAR 'Superterp'. Event #4 was measured at 15:22:33 UTC. new: **There was lightning activity detected at a distance of about 100 km from the Superterp. The shower passed through the edge of a rather extended cloud system.**

| Calculation | I* | | | II | | | III | | |
|---|---|---|---|---|---|---|---|---|---|
| Energy (eV) | $4.5 \times 10^{16}$ | | | $3.3 \times 10^{16}$ | | | $2.3 \times 10^{16}$ | | |
| Layer | $h$ | $E$ | $\alpha$ | $h$ | $E$ | $\alpha$ | $h$ | $E$ | $\alpha$ |
| 0 | 15.0 | 30 | 158 | 15.0 | 30 | 155 | 15.0 | 30 | 146 |
| 1 | 7.7 | 43 | 56 | 7.4 | 46 | 58 | 6.8 | 53 | 62 |
| 2 | 3.7 | -31 | -5 | 3.5 | -37 | 6 | 3.4 | -31 | 16 |
| 3 | 2.2 | 22 | -95 | 2.3 | 16 | -99 | 2.0 | 15 | -99 |
| $X_{\mathrm{max}}$ (g/cm$^2$) | 540 | | | 580 | | | 633 | | |
| $X_{\mathrm{max}}$ (km) | 5.7 | | | 5.2 | | | 4.6 | | |
| $\chi^2_{3D}$ | 1.53 | | | 1.70 | | | 2.14 | | |
| $\chi^2_C$ | 3.2 | | | 3.6 | | | 5.5 | | |
| $f_r$ | 8 | | | 12 | | | 24 | | |

**Table S4.** Same as Table S1 but for event #4. **new: We noted that the chi-square improved by more than 1 unit when allowing for an electric field above the cloud. We have selected calculation I as the preferred one because it has the smallest value for $f_r$.**

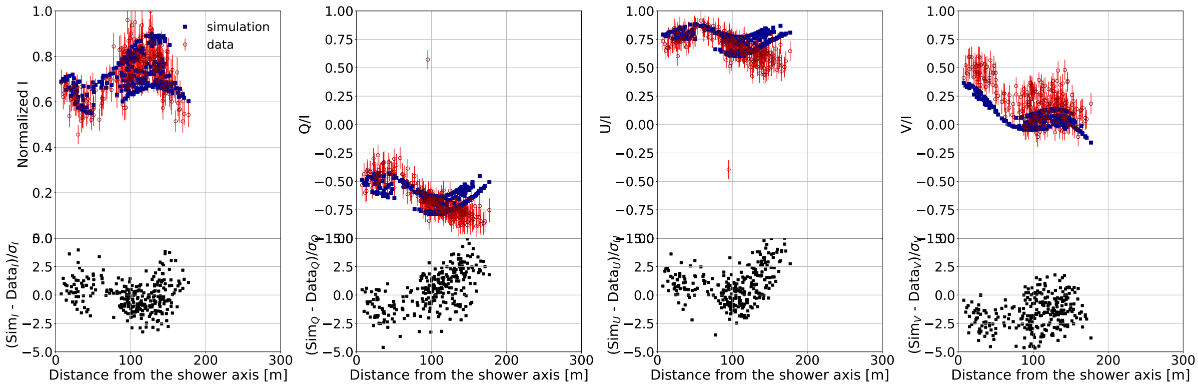


**Figure S8.** Same as Fig. S4 but for event #4 where simulation I is selected. **new: The ring-like intensity structure indicates a strong destructive interference between the emissions from different layers. The rather large value of the circular polarization near the core is a sign that the fields in different layers make a twist.**

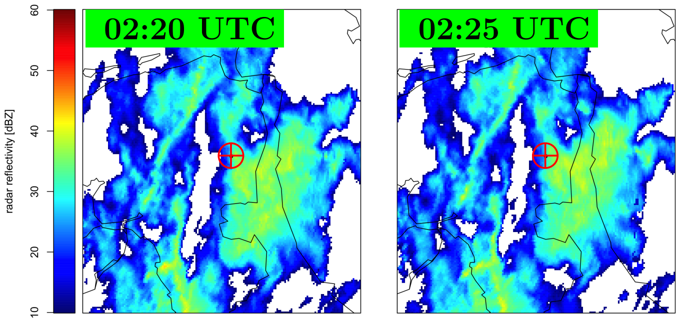


**Figure S9.** The red ⊕ marks the location of the LOFAR 'Superterp'. Event #5 was measured at 02:20:21 UTC. **new: The extensive cloud was not moving much and the shower passed through its edge. There was no lightning activity detected.**

| Calculation | I* | | | II | | | III | | |
|---|---|---|---|---|---|---|---|---|---|
| Energy (eV) | $2.4 \times 10^{16}$ | | | $2.0 \times 10^{16}$ | | | $1.5 \times 10^{16}$ | | |
| Layer | $h$ | $E$ | $\alpha$ | $h$ | $E$ | $\alpha$ | $h$ | $E$ | $\alpha$ |
| 1 | 7.0 | 72 | -53 | 7.1 | 65 | -45 | 7.1 | 72 | -39 |
| 2 | 5.5 | 104 | -106 | 5.3 | 106 | -107 | 5.2 | 106 | -107 |
| 3 | 3.2 | 16 | -168 | 3.3 | 14 | -170 | 3.3 | 13 | -173 |
| $X_{\max}$ (g/cm$^2$) | 520 | | | 585 | | | 640 | | |
| $X_{\max}$ (km) | 6.0 | | | 5.2 | | | 4.5 | | |
| $\chi^2_{3D}$ | 0.86 | | | 0.86 | | | 0.87 | | |
| $\chi^2_C$ | 1.02 | | | 1.13 | | | 1.09 | | |
| $f_r$ | 3 | | | 5 | | | 8 | | |

**Table S5.** Same as Table S1 but for event #5. **new: We have chosen I this as the preferred result because the norm factor is smallest while in addition it has a good value for the reduced $\chi^2$.**

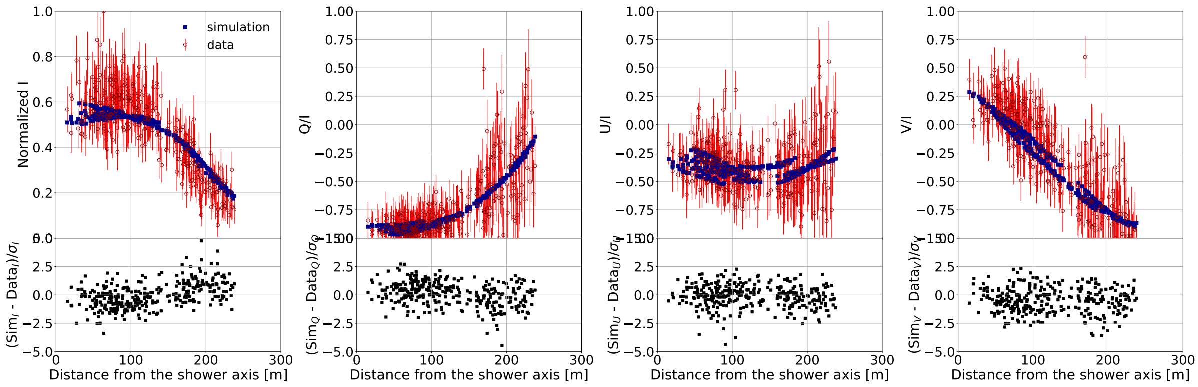


**Figure S10.** Same as Fig. S6 but for event #5 where simulation I is selected. The core position was moved by 15 m from the original location determined from the LORA data. new: **The intensity has a clear maximum at the core position indicating that there is little destructive interference and thus that the electric fields all point in basically the same direction, as is seen indeed from Table S5. The pattern for the circular polarization is similar to that of a fair-weather event. The direction of the linear polarization is however orthogonal to what one would expect for fair weather ($Q/I$=-1 in stead of +1).**

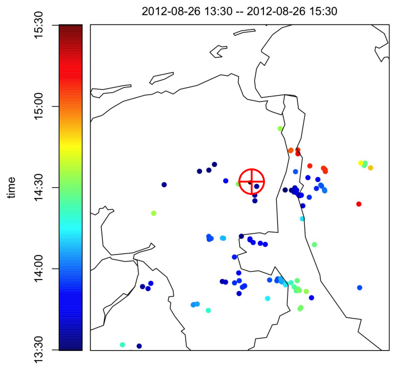


**Figure S11.** Lightning discharges on 26/08/2012 between 13:30 and 15:00 UTC. The red ⊕ gives the location of LOFAR 'Superterp'. There were three events measured on this day. Event #6 was measured at 13:52:23 UTC, event #7 at 14:02:56 UTC, and event #8 at 14:28:19 UTC.

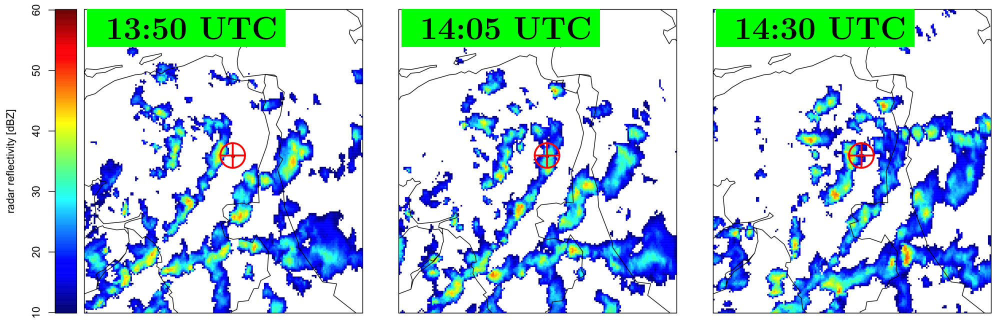


**Figure S12.** The red ⊕ in the center marks the location of the LOFAR 'Superterp'. There were 3 events measured on this day. Event #6 was measured at 13:52:23 UTC, event #7 at 14:02:56 UTC, and event #8 at 14:28:19 UTC. new: **While events #6 and #7 passed through different sides of the same cloud, event #8 passed through a different one. The clouds were moving rather fast from west to east.**

| Calculation | I* | | | II | | | III | | |
|---|---|---|---|---|---|---|---|---|---|
| Energy (eV) | $4.4 \times 10^{16}$ | | | $2.6 \times 10^{16}$ | | | $1.8 \times 10^{16}$ | | |
| Layer | $h$ | $E$ | $\alpha$ | $h$ | $E$ | $\alpha$ | $h$ | $E$ | $\alpha$ |
| 1 | 9.1 | 57 | -63 | 8.7 | 75 | -60 | 9.1 | 86 | -57 |
| 2 | 4.0 | 3 | -154 | 4.1 | 6 | -157 | 4.0 | 14 | 165 |
| 3 | 1.2 | 4 | -20 | 0.2 | 44 | 3 | 1.9 | 7 | -65 |
| $X_{\text{max}}$ (g/cm$^2$) | 550 | | | 602 | | | 700 | | |
| $X_{\text{max}}$ (km) | 5.6 | | | 5.0 | | | 3.8 | | |
| $\chi^2_{3D}$ | 1.08 | | | 1.09 | | | 1.06 | | |
| $\chi^2_C$ | 2.02 | | | 2.12 | | | 3.19 | | |
| $f_r$ | 3 | | | 9 | | | 25 | | |

**Table S6.** Same as Table S1 but for event #6. new: **Calculation I is selected because of the agreement between the results of the CoREAS and the MGMR3D calculation and the good value for the norm factor $f_r$.**

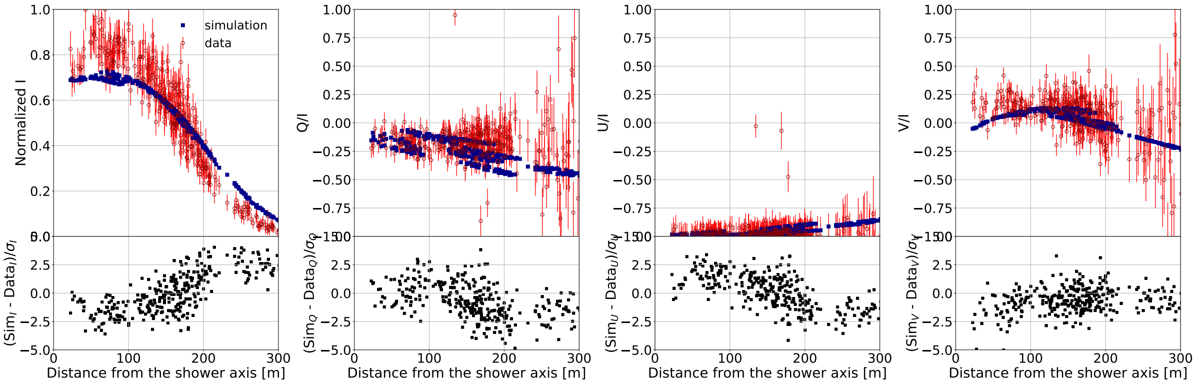


**Figure S13.** Same as Fig. S6 but for event #6 where simulation I is selected. The core position was moved by 10 m from the original location determined from the LORA data. new: **The intensity and circular polarization patterns are reminiscent of a fair weather event with a small dent in the intensity near the core, however the linear polarization is at 45 degree ($Q/I$=0 in stead of +1). This indicates moderate destructive interference and an electric field that is perpendicular to the of the geomagnetic force.**

| Calculation | I | | | II* | | | III | | |
|---|---|---|---|---|---|---|---|---|---|
| Energy (eV) | $5.9 \times 10^{16}$ | | | $3.9 \times 10^{16}$ | | | $3.7 \times 10^{16}$ | | |
| Layer | $h$ | $E$ | $\alpha$ | $h$ | $E$ | $\alpha$ | $h$ | $E$ | $\alpha$ |
| 1 | 5.7 | 27 | -29 | 5.8 | 30 | -29 | 5.6 | 38 | -30 |
| 2 | 3.5 | 80 | 181 | 3.4 | 83 | 180 | 3.3 | 82 | 177 |
| 3 | 1.4 | 27 | 22 | 1.7 | 13 | 30 | 1.6 | 15 | 15 |
| $X_{\mathrm{max}}$ (g/cm$^2$) | 550 | | | 650 | | | 700 | | |
| $X_{\mathrm{max}}$ (km) | 5.4 | | | 4.1 | | | 3.6 | | |
| $\chi^2_{3D}$ | 2.62 | | | 2.11 | | | 1.95 | | |
| $\chi^2_C$ | 2.06 | | | 1.95 | | | 2.73 | | |
| $f_r$ | 8 | | | 8 | | | 8 | | |

**Table S7.** Same as Table S1 but for event #7. new: **The fields in layers 1 and 2 cause a strong destructive interference which is responsible for the ring structure in the intensity.**

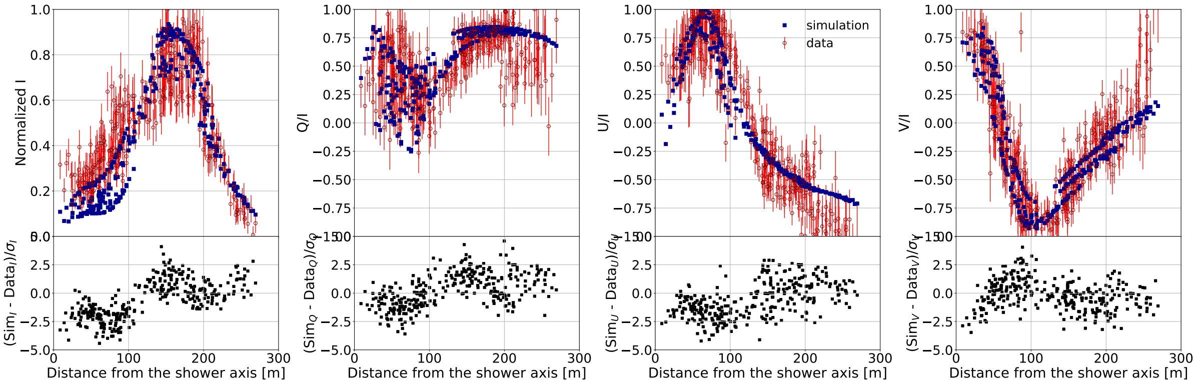


**Figure S14.** Same as Fig. S6 but for event #7 where simulation II is selected. The core position was moved by 30 m from the original location determined from the LORA data. new: **The footprint is rather complicated with a ring in intensity with a large radius, indicative of interference of two high-lying layers, and a large circular polarization near the core, indicating that one layer has a force that is not aligned.**

| Calculation | I | | | II* | | | III | | |
|---|---|---|---|---|---|---|---|---|---|
| Energy (eV) | $2.3 \times 10^{16}$ | | | $2.5 \times 10^{16}$ | | | $1.7 \times 10^{16}$ | | |
| Layer | $h$ | $E$ | $\alpha$ | $h$ | $E$ | $\alpha$ | $h$ | $E$ | $\alpha$ |
| 1 | 6.9 | 61 | -98 | 7.2 | 42 | -97 | 8.3 | 44 | -94 |
| 2 | 3.5 | 94 | -147 | 3.7 | 73 | -137 | 3.7 | 92 | -136 |
| 3 | 2.9 | 46 | 64 | 3.0 | 24 | 69 | 3.2 | 19 | 71 |
| $X_{\max}$ (g/cm$^2$) | 580 | | | 656 | | | 710 | | |
| $X_{\max}$ (km) | 5.4 | | | 4.4 | | | 3.8 | | |
| $\chi^2_{3D}$ | 2.06 | | | 1.96 | | | 1.89 | | |
| $\chi^2_C$ | 3.22 | | | 2.88 | | | 2.77 | | |
| $f_r$ | 2 | | | 1 | | | 3 | | |

**Table S8.** Same as Table S1 but for event #8.

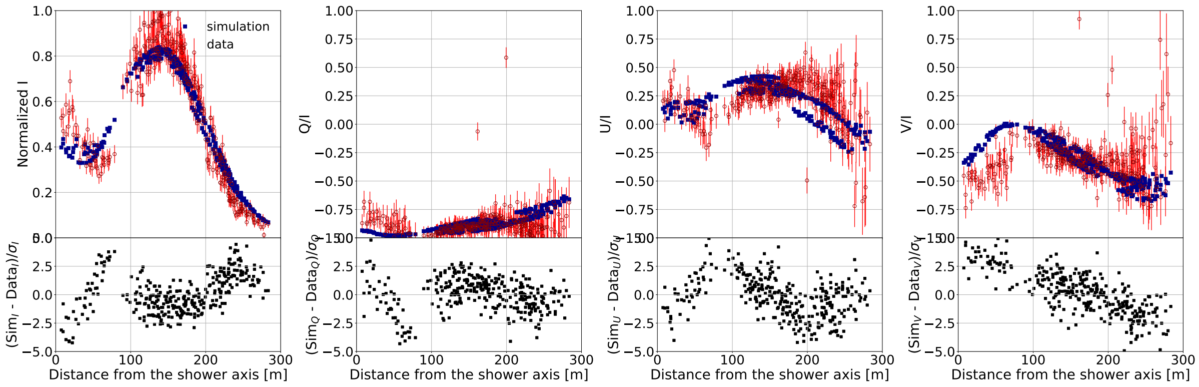


**Figure S15.** Same as Fig. S6 but for event #8 where simulation II is selected. new: **The ring like structure shows that there is a considerable amount of destructive interference with fields in the $\mathbf{v}\times(\mathbf{v}\times\mathbf{B})$ direction since $Q/I = -1$.**

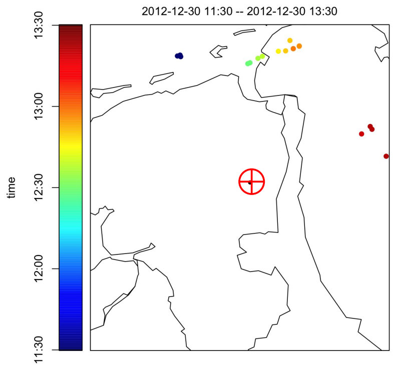


**Figure S16.** Lightning discharges on 30/12/2012. The red ⊕ is the location of LOFAR 'Superterp'. Event #9 was measured at 12:38:37 UTC.

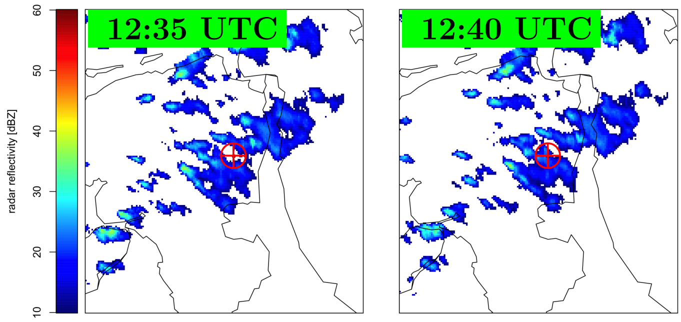


**Figure S17.** The red ⊕ marks the location of the LOFAR 'Superterp'. Event #9 was measured at 12:38:37 UTC. **new: Fig. S16 shows that the nearest lightning activity was detected at a distance of 100 km.**

| Calculation | I | | | II* | | | III | | |
|---|---|---|---|---|---|---|---|---|---|
| Energy (eV) | $2.8 \times 10^{17}$ | | | $2.4 \times 10^{17}$ | | | $1.4 \times 10^{17}$ | | |
| Layer | $h$ | $E$ | $\alpha$ | $h$ | $E$ | $\alpha$ | $h$ | $E$ | $\alpha$ |
| 1 | 4.6 | 34 | 117 | 4.6 | 34 | 116 | 4.8 | 34 | 115 |
| 2 | 1.4 | 43 | 25 | 1.5 | 33 | 21 | 1.5 | 26 | 19 |
| $X_{\mathrm{max}}$ (g/cm$^2$) | 670 | | | 720 | | | 773 | | |
| $X_{\mathrm{max}}$ (km) | 3.8 | | | 3.2 | | | 2.7 | | |
| $\chi^2_{3D}$ | 2.0 | | | 2.0 | | | 2.2 | | |
| $\chi^2_C$ | 13. | | | 6.5 | | | 6.4 | | |
| $f_r$ | 3 | | | 3 | | | 7 | | |

**Table S9.** Same as Table S1 but for event #9. new: **This case could be fitted well with a simple 2 layer structure. The extracted strengths depend strongly on $X_{\mathrm{max}}$ since it lies at an height close to $h_3$. Variation of $X_{\mathrm{max}}$ thus requires a sizable compensation in the electric field to keep similar currents. Simulation II is preferred based on the reduced $\chi^2$ as well as the ratio $f_r$.**

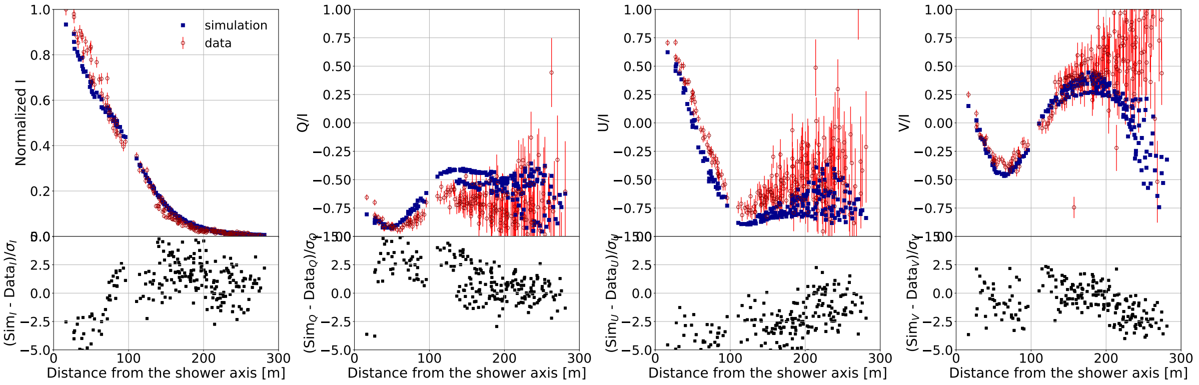


**Figure S18.** Same as Fig. S6 but for event #9 where simulation II is selected. The core position was moved by 10 m from the original location determined from the LORA data.

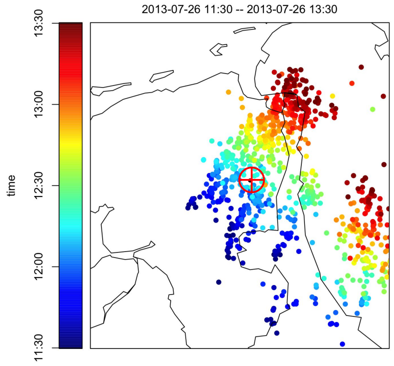


**Figure S19.** Lightning discharges on 26/07/2013. The red ⊕ is the location of LOFAR 'Superterp'. Event #10 was measured at 12:17:26 UTC. new: **One can see the lightning storm moving from south-west to north-east where at the time of the cosmic ray event it was overhead of the Superterp.**

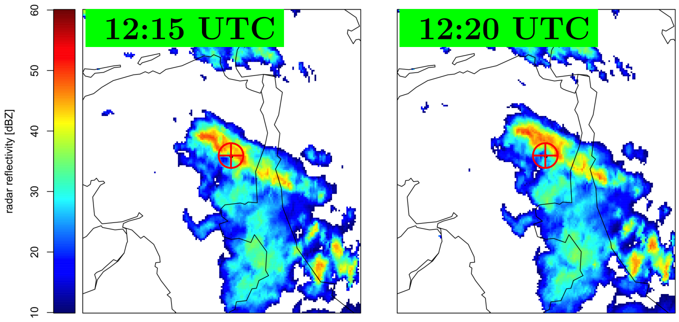


**Figure S20.** The red ⊕ marks the location of the LOFAR 'Superterp'. Event 10 was measured at 12:17:26 UTC. new: **Since this is a real thundercloud with lightning activity one expects to see much larger values for the radar-reflectivity, which is indeed the case.**

| Calculation | I* | | | II | | | III | | |
|---|---|---|---|---|---|---|---|---|---|
| Energy (eV) | $3.9 \times 10^{16}$ | | | $3.5 \times 10^{16}$ | | | $1.3 \times 10^{16}$ | | |
| Layer | $h$ | $E$ | $\alpha$ | $h$ | $E$ | $\alpha$ | $h$ | $E$ | $\alpha$ |
| 1 | 7.4 | 88 | 80 | 7.0 | 54 | 81 | 6.2 | 75 | 78 |
| 2 | 5.0 | 92 | -102 | 4.6 | 95 | -103 | 4.4 | 82 | -104 |
| 3 | 3.5 | 68 | -136 | 3.7 | 35 | -140 | 3.5 | 28 | -140 |
| $X_{\max}$ (g/cm$^2$) | 560 | | | 630 | | | 700 | | |
| $X_{\max}$ (km) | 5.1 | | | 4.3 | | | 3.5 | | |
| $\chi^2_{3D}$ | 1.15 | | | 1.15 | | | 1.21 | | |
| $\chi^2_C$ | 1.46 | | | 2.80 | | | 2.82 | | |
| $f_r$ | 9 | | | 14 | | | 127 | | |

**Table S10.** Same as Table S1 but for event #10.

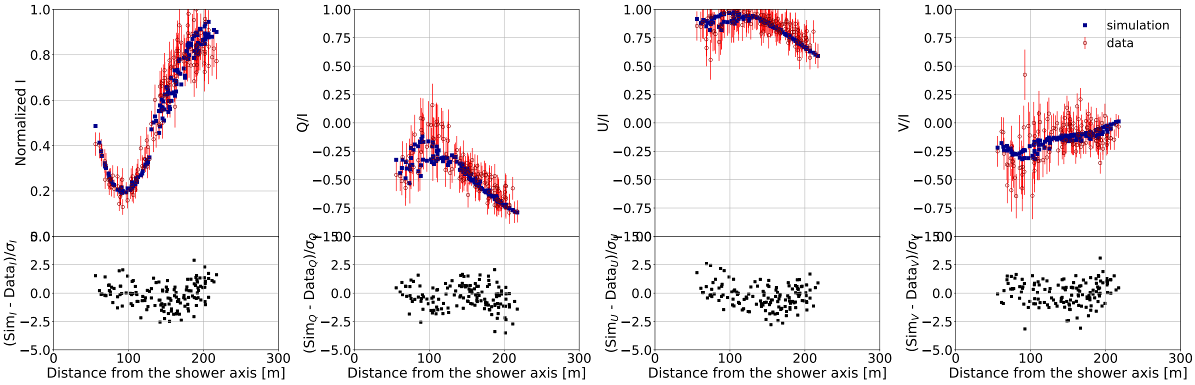


**Figure S21.** Same as Fig. S6 but for event #10 where simulation I is selected. new: **Based on the intensity footprint one expects to have a strong destructive interference between different layers. The polarization indicates that the fields are at a large angle with v×B.**

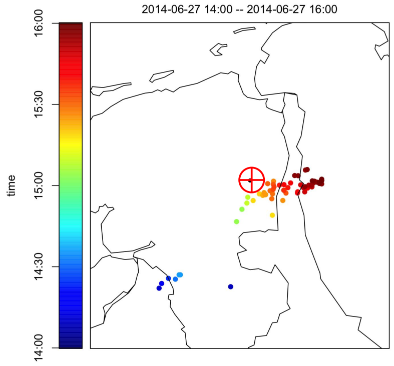


**Figure S22.** Lightning discharges on 27/06/2014. The red ⊕ gives the location of the LOFAR 'Superterp'. Event #11 was measured at 14:44:03 UTC. new: **A small active core is moving over the Superterp right at the time of the shower observation.**

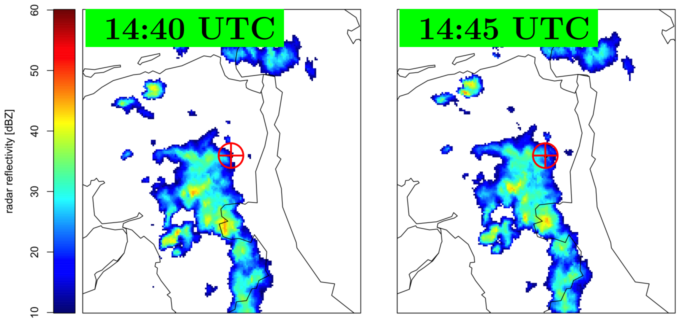


**Figure S23.** The red ⊕ marks the location of the LOFAR 'Superterp'. Event #11 was measured at 14:44:03 UTC. new: **The shower passes through the side of a rather large cloud where the active lightning core lies close to the Superterp.**

| Calculation | I | | | II* | | | III | | |
|---|---|---|---|---|---|---|---|---|---|
| Energy (eV) | $3.7 \times 10^{16}$ | | | $2.6 \times 10^{16}$ | | | $2.1 \times 10^{16}$ | | |
| Layer | $h$ | $E$ | $\alpha$ | $h$ | $E$ | $\alpha$ | $h$ | $E$ | $\alpha$ |
| 1 | 6.2 | 84 | 43 | 6.4 | 104 | 41 | 6.7 | 94 | 40 |
| 2 | 4.5 | 57 | -27 | 4.5 | 60 | -28 | 4.2 | 60 | -28 |
| 3 | 3.0 | 4 | -113 | 3.0 | 4 | -115 | 3.0 | 5 | -137 |
| $X_{\max}$ (g/cm$^2$) | 560 | | | 618 | | | 710 | | |
| $X_{\max}$ (km) | 5.1 | | | 4.6 | | | 3.3 | | |
| $\chi^2_{3D}$ | 0.78 | | | 0.78 | | | 0.83 | | |
| $\chi^2_C$ | 3.01 | | | 2.99 | | | 3.06 | | |
| $f_r$ | 1 | | | 2 | | | 3 | | |

**Table S11.** Same as Table S1 but for event #11. new: **Simulation II is preferred because the ratio $f_r$ is close to unity.**

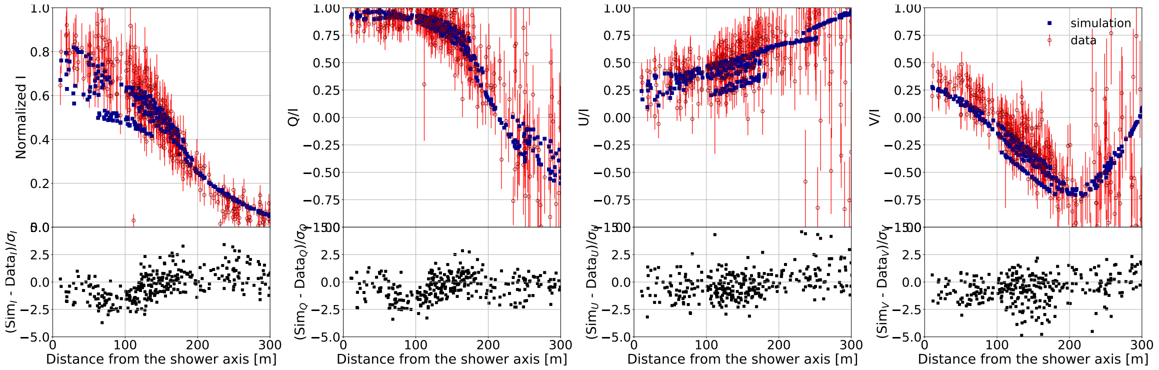


**Figure S24.** Same as Fig. S6 but for event #11 where simulation II is selected. new: **Since at large distances the footprint deviates most strongly from the fair-weather case one expects strong fields in the high layers.**
